# Linker Design Principles for the Precision Targeting of Oncogenic G-Quadruplex DNA with G4-Ligand-Conjugated Oligonucleotides

**DOI:** 10.1021/acs.bioconjchem.5c00008

**Published:** 2025-03-20

**Authors:** Alva Abrahamsson, Andreas Berner, Justyna Golebiewska-Pikula, Namrata Chaudhari, Emelie Keskitalo, Cecilia Lindgren, Marcin K. Chmielewski, Sjoerd Wanrooij, Erik Chorell

**Affiliations:** † Department of Chemistry, Umeå University, SE-901 87 Umeå, Sweden; ‡ Departments of Medical Biochemistry and Biophysics, Umea University, SE-907 36 Umeå, Sweden; § Institute of Bioorganic Chemistry, Polish Academy of Sciences, Noskowskiego 12/14, 61-704 Poznan, Poland; ∥ FutureSynthesis, sp. z o.o., ul. Rubież 46B, 61-612 Poznan, Poland

## Abstract

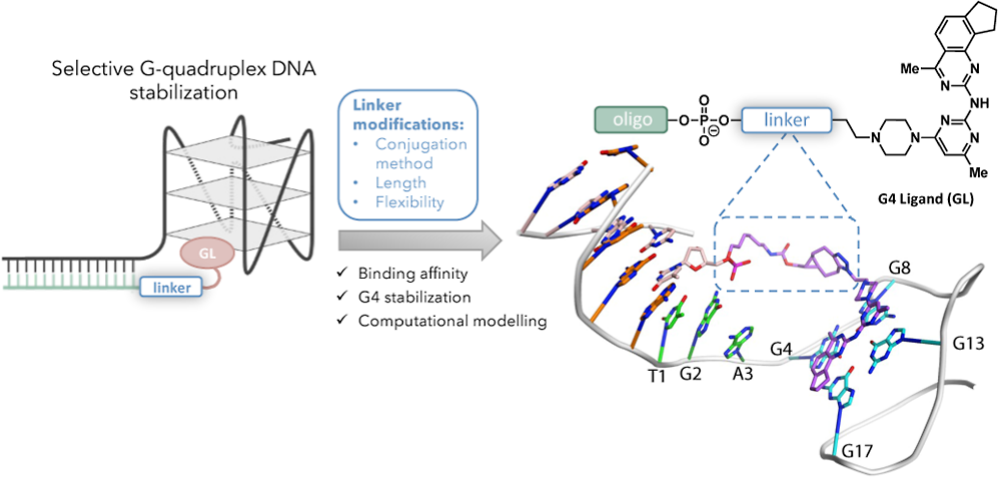

G-quadruplex (G4) DNA structures are noncanonical secondary structures found in key regulatory regions of the genome, including oncogenic promoters and telomeres. Small molecules, known as G4 ligands, capable of stabilizing G4s hold promise as chemical probes and therapeutic agents. Nevertheless, achieving precise specificity for individual G4 structures within the human genome remains a significant challenge. To address this, we expand upon G4-ligand-conjugated oligonucleotides (GL-Os), a modular platform combining the stabilizing properties of G4-ligands with the sequence specificity of guide DNA oligonucleotides. Central to this strategy is the linker that bridges the G4 ligand and the guide oligonucleotide. In this study, we develop multiple conjugation strategies for the GL-Os that enabled a systematic investigation of the linker in both chemical composition and length, enabling a thorough assessment of their impact on targeting oncogenic G4 DNA. Biophysical, biochemical, and computational evaluations revealed GL-Os with optimized linkers that exhibited enhanced binding to target G4s, even under thermal or structural stress. Notably, longer linkers broadened the range of targetable sequences without introducing steric hindrance, thereby enhancing the platform’s applicability across diverse genomic contexts. These findings establish GL-Os as a robust and versatile tool for the selective targeting of individual G4s. By facilitating precise investigations of G4 biology, this work provides a foundation for advancing G4-targeted therapeutic strategies and exploring their role in disease contexts.

## Introduction

G-quadruplex (G4) DNA structures are secondary motifs formed in guanine-rich single-stranded DNA. All G4 structures share a central core composed of four guanines, known as G-tetrads, which self-stack and are stabilized by monovalent cations such as K^+^ or Na^+^.^1^ G4s are polymorphic and can adopt various topologies with different strand orientations.^2^ Despite these variations, the core structure with the stacked G-tetrads is a consistent feature among all G4s.

In the human genome, G4 structures are associated with the regulation of cellular processes such as telomere extension by the telomerase and stalling of the replication fork during DNA replication.^3,4^ Furthermore, G4 structures are abundant in promoter regions where they are involved in transcriptional regulation.^5−7^ Guanine-rich promoter regions, such as the nuclease hypersensitive element (NHEIII) or Pu27 of the *c-MYC* oncogene, have been shown to form a G4 structure that is highly linked with the regulation of the MYC protein.^8−12^ The transcription factor MYC is estimated to be dysregulated in up to 70% of human cancer types, making it an attractive drug target.^12−14^ While small molecules targeting the MYC protein are challenging to develop, G4 structures in the *MYC* promoter region represent a promising therapeutic target with the potential to instead modulate the expression of the MYC protein.^5,15^

In recent decades, numerous small organic molecules have been developed to bind and stabilize G4 DNA structures.^5,16−19^ However, only a few have advanced into clinical trials.^20,21^ The main challenge lies in the selectivity and the G4-ligand’s inability to differentiate between G4 structures formed in different sequences.^22^ This difficulty is likely due to the shared core structure of all G4s, which serve as the primary binding site for small molecules. With over 700,000 sequences in the human genome having the potential to form G4 structures,^6,7,23,24^ achieving intra-G4 selectivity is crucial to both study the central functions of individual G4 structures and probe their therapeutic potential as drug targets.

**Figure 1:**
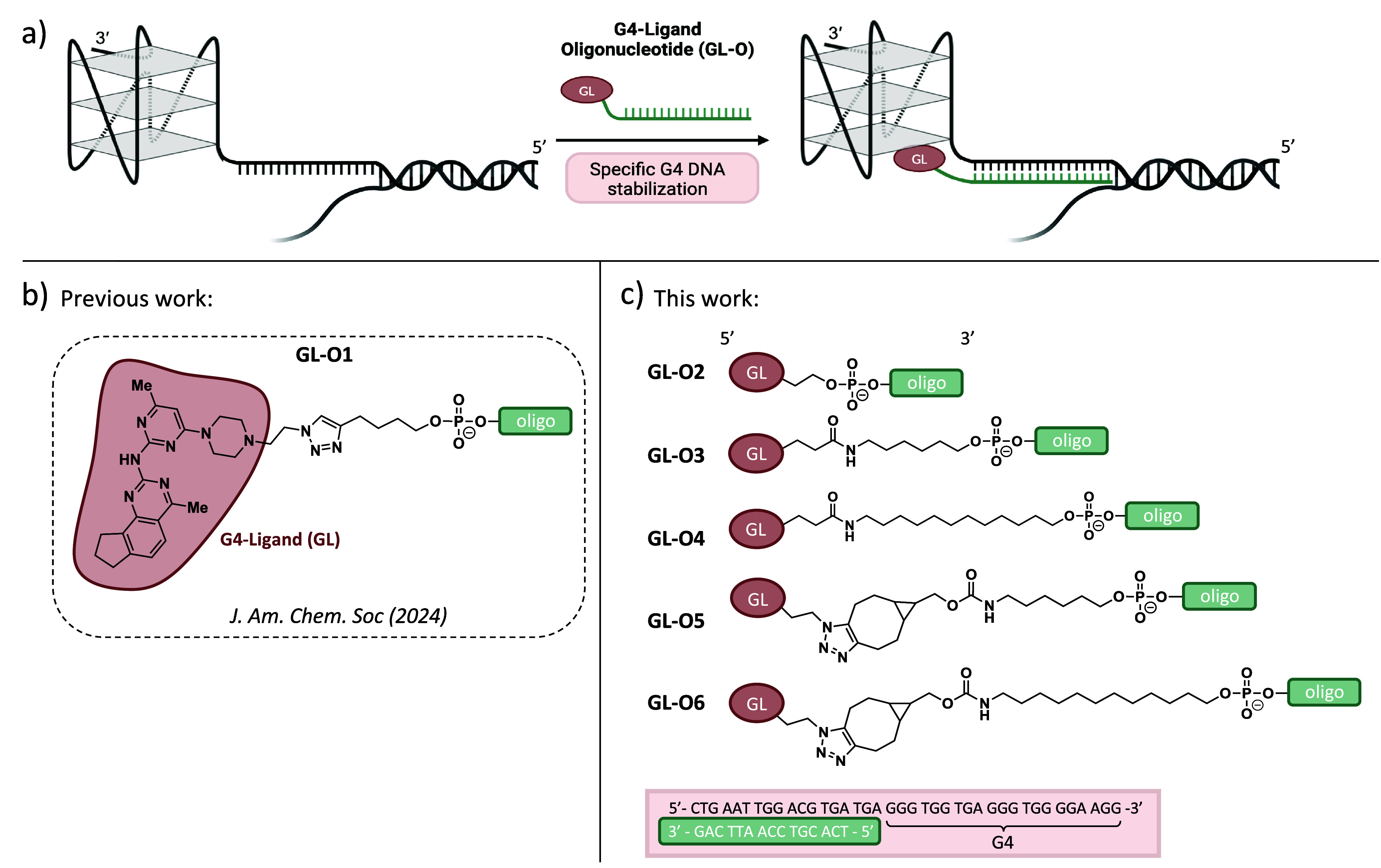
Overview of the G4-ligand-conjugated oligonucleotide (GL-O) strategy and GL-Os synthesized and discussed in this study. (a) Schematic representation of the G4-ligand-conjugated oligonucleotide (GL-O) strategy developed to specifically stabilize individual G4 DNA structures. (b) Chemical structure of the first developed GL-O with the structure of the G4-ligand (GL) and linker.^25^ (c) Outline of the GL-Os described in this study and sequence of the G4 target used to assess the different linker-modified GL-Os.

To overcome the selectivity obstacle, we recently described an approach called G4-ligand-conjugated oligonucleotides (GL-Os).^25^ The GL-O modality consists of a G4-ligand conjugated to a guide DNA oligonucleotide that is complementary to the sequence flanking the target G4 (Figure 1a). Introducing the guide oligonucleotide as recognition motifs enabled selective binding and stabilization of only the target G4 with the complementary flanking sequence.^25^ Furthermore, the GL-O strategy provides great flexibility in G4 targets by simply replacing the guide oligonucleotide.

In the reported GL-O modality, the G4-ligand was linked to the oligonucleotide through copper-catalyzed azide–alkyne cycloaddition (CuAAC) (Figure 1b). This established conjugation method produces a small triazole linkage; however, phosphorothioate backbone oligonucleotides are not compatible with the solution-phase CuAAC method. Phosphorothioate modifications are widely used to enhance oligonucleotide stability under cellular and in vivo conditions, making it essential for conjugation methods to be compatible with this modification. To expand the applicability of the GL-Os in cellular experimental setups, we therefore aimed to find conjugation methods that allow for diverse oligonucleotide modifications to be introduced to the GL-O. Crucially, this approach would allow optimization of the linker between the two recognition motifs in the GL-O strategy without compromising the selectivity, as already demonstrated in our previous work.^25^

Numerous alternative solution-phase conjugation methods for oligonucleotides are available, such as strain-promoted azide–alkyne cycloaddition (SPAAC) and amide couplings.^26−28^ These methods provide covalent and stable linkages and are generally compatible with various oligonucleotide modifications. However, implementing a new conjugation method for the GL-O modality involves changing the chemical properties of the linker, which potentially can alter the binding and stabilization of the target G4. Furthermore, the linker length likely plays a significant role in facilitating the interaction between the G4 ligand and the G4 structure. Previous data suggests that there may be some flexibility in the linker design;^25^ however, a more extensive assessment of the linker’s impact needs to be conducted.

To explore and broaden the scope of the GL-O strategy, we developed methods herein to construct novel classes of GL-Os by conjugating the G4-ligand with oligonucleotides in solution through SPAAC and amide coupling reactions. Additionally, we converted the ligand into a phosphoramidite to incorporate it directly on the 5′-end using solid-phase oligonucleotide synthesis (SPOS). Combined, this generated a set of new GL-Os with both different linker design and linker lengths (Figure 1c, see sequences in Table S2). The impact of these variations on G4 binding and stabilization was evaluated using biochemical and biophysical assays: (1) microscale thermophoresis (MST) to determine binding affinity, (2) proton nuclear magnetic resonance (^1^H NMR) spectroscopy to study the binding interactions, (3) ^1^H NMR melting experiments to evaluate G4 stabilization, and (4) a DNA polymerase stop assay to examine G4 stabilization under conditions resembling a biological setting. We further evaluated how the G4 binding and stabilization of GL-Os with varying linker lengths and chemical compositions were influenced by changes in the distance between the guide oligonucleotide binding sequence of GL-O and the target G4 structure. To gain molecular-level insight into complementing these experimental data, we used computational modeling. Overall, the study expanded the GL-O platform and uncovered key information about the linker design, which will be essential in the future development of GL-Os to explore the functions of individual G4 structures and their therapeutic potential. In addition, these findings can benefit similar approaches as well as oligonucleotide-based strategies in general.

## Experimental Procedure

### Safety Statement

The work conducted during this project adhered to standard laboratory safety protocols and procedures. No unexpected safety situations or hazards were encountered, and the materials and methods employed did not require any precautions beyond routine laboratory safety measures. Proper handling, storage, and disposal practices were followed throughout the project to ensure a safe working environment.

### General Procedure for G4-Ligand Conjugation of Oligonucleotides in Solution

(Detailed procedures for the preparation of each GL-O can be found in the Supporting Information.) Oligo (equipped with different 5́-functional groups) was dissolved in a 1 mM solution in Milli-Q water or 0.1 M NaHCO_3_ (aq) in an Eppendorf tube. Subsequently, freshly prepared reagents and catalysts were added, along with the G4-ligand containing either an azide or carboxylic acid, and the reaction was vortexed and allowed to shake at room temperature overnight. Upon completion, the reaction was either quenched or precipitated before the next step or further diluted with water before filtering with a 45 μm syringe filter. Purification was done on RP-HPLC using a C18 semipreparative column with a 2 mL/min flow rate and a linear gradient of 5 to 60% solvent B (100% ACN). Solvent A was a 50 mM triethylammonium acetate buffer, pH 7. The conjugated product was confirmed with HRMS.

### G4-Ligand Conjugation of Oligonucleotides on Solid Support

The G4-ligand was converted into a phosphoramidite, dissolved in ACN/DCM (1:1 v/v) to a 0.15 M solution, and loaded onto the synthesizer along with the other DNA phosphoramidites. Synthesis was done on an automated oligonucleotide synthesizer (1 μmol scale) using a CPG solid support. The detritylation step was done using 3% dichloroacetic acid in DCM, activation by 0.25 M 4,5-dicyanoimidazole in ACN, oxidation using 0.05 M iodine in 90% pyridine, and capping using equal volumes of solution A (20% *N*-methylimidazole in ACN) and solution B (30% lutidine, 20% acetic anhydride in ACN). The phosphoramidite G4-ligand was activated by 0.25 M 5-benzylmercaptotetrazole and coupled onto the 5́-end of the oligonucleotide. Cleavage and deprotection were done in 30% ammonia for 24 h at ambient temperature followed by evaporation in Speedvac and purification on RP-HPLC. The conjugated product was confirmed by HRMS.

### Microscale Thermophoresis

G4 DNA was 5́-labeled with Cy5 and consisted of the G4-forming sequence and 5′ flanking sequence. The G4 DNA was annealed in MST buffer (10 mM potassium phosphate, 100 mM KCl, 0.05% Tween 20, pH 7.4) by heating at 96 °C for 5 min followed by cooling to room temperature before storing in a fridge overnight. All MST experiments were performed on a Monolith NT.115 (Nanotemper, Germany) instrument and performed in MST buffer. The G4 DNA concentration was kept constant at 10–20 nM, to which GL-Os were serially diluted (1:1) with the highest concentration of 5–20 μM. MST traces and binding affinity constants (*K*_D_) were obtained using the Monolith analysis software and plotted and visualized in GraphPad Prism 10.

### Nuclear Magnetic Resonance Spectroscopy and Thermal Stabilization

G4 DNA solution was annealed in 10 mM potassium phosphate buffer (3 mM KCl, 110 μM G4 DNA, pH 7.4) by heating at 96 °C for 5 min followed by cooling down slowly to room temperature and storing in a fridge overnight. To the G4 DNA solution, 10% D_2_O was added to yield a 100 μM solution which was transferred to a 3 mm NMR tube. GL-O or oligo (1 mM in H_2_O) was added (2 μL, 0.5 equiv), and after 10 min, ^1^H NMR spectrum was recorded. This was repeated one more time with an additional 0.5 equiv of GL-O to yield a 1:1 molar ratio with the G4 DNA. The instrument setting used was an 850 MHz AVANCE III HD spectrometer equipped with a 5 mm TCI cryoprobe at 298 K. Transmitter frequency offset (O1P) was set at 4.7 ppm, and the spectral width was fixed as 22 ppm. Excitation sculpting was used in the 1D ^1^H experiments, and 512 scans were used to record the spectra. For the thermal stability assay, the samples were equilibrated at temperatures ranging from 313 to 338 K for 5 min followed by recording of the spectra every 2.5–10 K interval. The data was processed in MestreNova 10.0.2.

### Taq Polymerase Stop Assay

The Taq polymerase stop assay was adapted from Jamrokovi et al.^17^ DNA templates were annealed to fluorescently labeled primers in 100 mM KCl by heating to 95 °C for 5 min followed by slow cooling to room temperature. The indicated compound concentrations were added to a 40 nM annealed template in 1× Taq Buffer (10 mM Tris–HCl, pH 8.8, 50 mM KCl, Thermo Fisher Scientific), 1.5 mM MgCl_2_, and 0.05 U/μL Taq Polymerase (Thermo Fisher Scientific). Samples were preincubated on ice (10 min), and reactions were initiated with the addition of dNTPS (100 μM) and transfer of the samples to 37 °C. After 15 min at 37 °C, reactions were stopped by the addition of equal volume of 2× stop solution (0.5% SDS, 25 mM EDTA, XC-Dye in Formamide) and separated on a 12% polyacrylamide Tris–Borate–EDTA gel containing 25% formamide and 8 M urea. Fluorescent signal was detected with a Typhoon Scanner (Amersham Biosciences). The intensity of the full-length band was quantified using Image Quant TL 10.2 software (GE Healthcare Life Sciences) and compared to sample without compound.

### Computational Modeling

(Detailed procedures for the preparation of each GL-O can be found in the Supporting Information.) In short, the models were based on the NMR structure (PDB: 2MGN1). Molecular dynamics (MD) simulations using the GROMACS^29^ simulation package were used to sample the conformational landscape of the G4 oligonucleotides. Molecular docking using Glide within the Schrödinger package^30−34^ was thereafter performed to select conformational states, followed by modeling of the connecting linker.

## Results and Discussion

### Design and Synthesis of Linker-Modified GL-Os

**Scheme 1:**
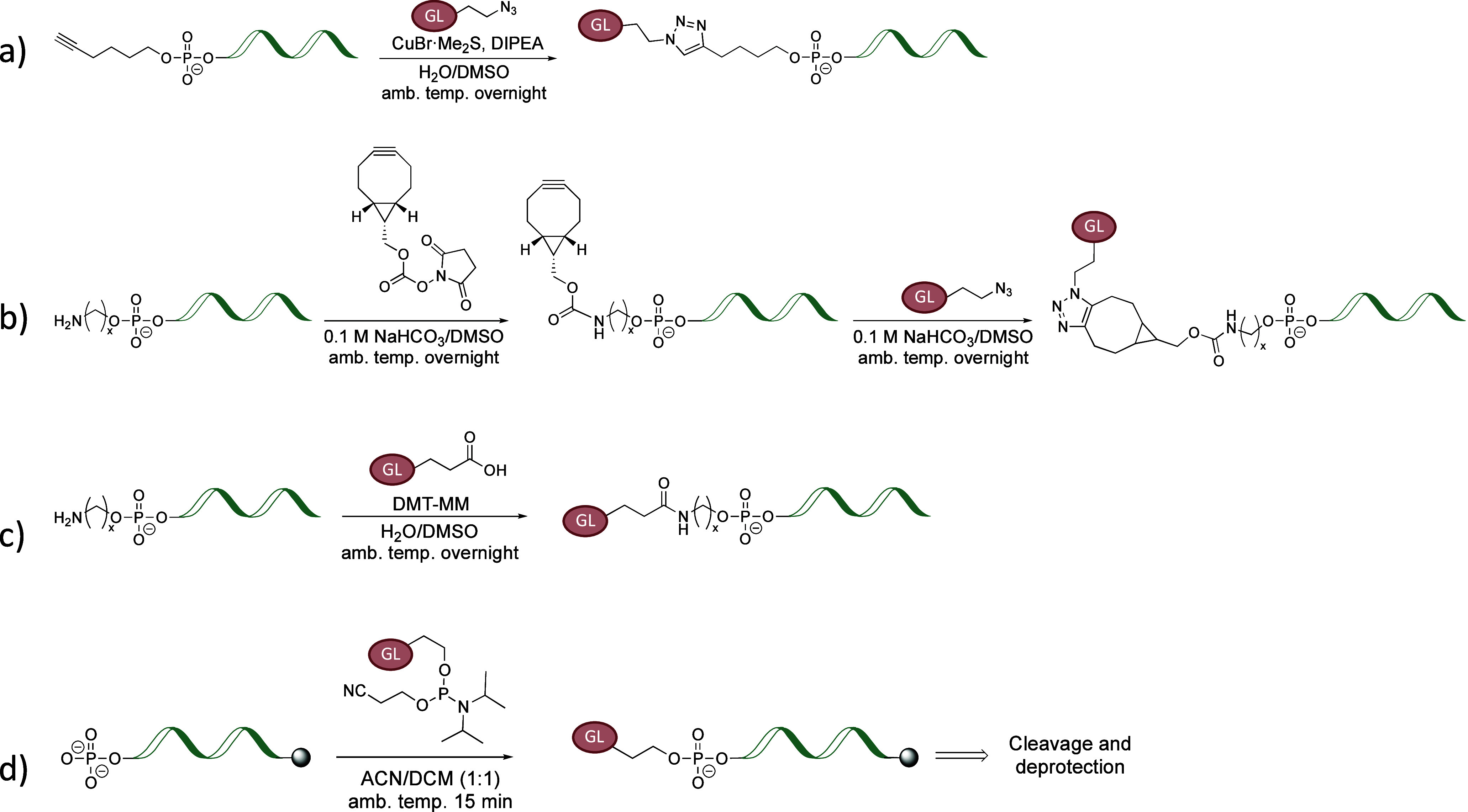
Conjugation of the G4-Ligand with Oligonucleotide through (a) Cu-Catalyzed Azide–Alkyne Cycloaddition, (b) Strain-Promoted Azide–Alkyne Cycloaddition, (c) Amide Coupling, and (d) Solid-Phase Oligonucleotide Synthesis

The initial GL-O design was based on the G4-ligand with a terminal azide that was conjugated to an oligonucleotide (15 nucleotide (nt)) containing a 5′-hexynyl group through CuAAC to give **GL-O1** (Scheme 1a and Table S2).^25^ To enhance the flexibility and broaden the application scope of the GL-O approach, we explored alternative conjugation methods and systematically evaluated how variations in the chemical composition and length of the linker influenced the GL-Os’ ability to bind and stabilize G4 structures.

**Scheme 2:**
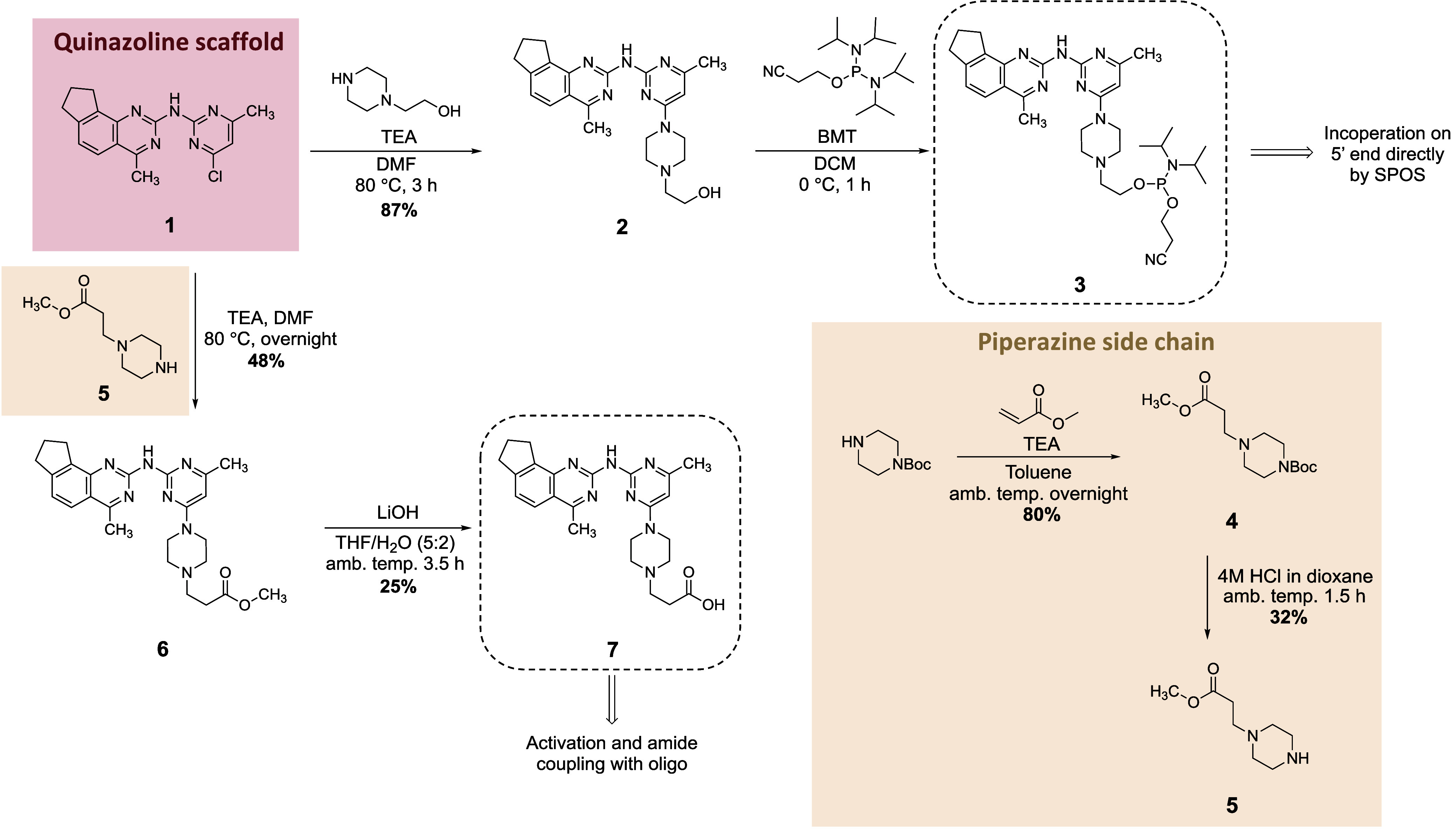
Synthesis of Modified G4-Ligands with a Phosphoramidite or Carboxylic Acid Handle for Further Conjugation

The GL-O with the shortest linker length was generated using SPOS to incorporate the G4-ligand to the 5́-end of the oligonucleotide using the standard phosphoramidate approach. The G4-ligand was first converted into a phosphoramidite by reacting the commercially available 1-(2-hydroxyethyl)piperazine with the quinazoline scaffold 1, yielding compound 2 in an 87% yield (Scheme 2). Compound 2 was then reacted with bis(diisopropylamino)(2-cyanoethoxy)phosphine in the presence of the activator 5-benzylmercaptotetrazole, producing target phosphoramidite 3. Due to instability, compound 3 was used immediately without further purification. The G4-ligand phosphoramidite was incorporated onto the 5́-end of the oligonucleotide using an automated synthesizer and SPOS (Scheme 1d). This gave **GL-O2** after cleavage from the solid support and deprotection of the base and backbone protecting groups using 30% ammonia (Figure 1c and Table S2).

To link the G4-ligand with the oligonucleotide using an amide coupling, the G4-ligand was synthesized with a terminal carboxylic acid (Scheme 2). The synthesis started from commercially available 1-boc-piperazine, which was reacted with methyl acrylate to give 4 in an 80% yield. Boc deprotection gave the piperazine methyl ester analogue 5 in a 32% yield, which was reacted with a quinazoline scaffold 1 to give compound 6 in a 48% yield. Basic hydrolysis of compound 6 produced the final carboxylic acid analogue 7 in a 25% yield. Conjugation of carboxylic acid analogue 7 with the oligonucleotide was achieved by activation of the carboxylic acid using coupling agent 4-(4,6-dimethoxy-1,3,5-triazin-2-yl)-4-methyl-morpholinium chloride (DMT-MM) for 15 min (Scheme 1c) followed by addition of the oligonucleotide with a 5́-end primary amine to give **GL-O3** (Figure 1c and Table S2).

The next conjugation method employed was SPAAC, which uses the previously described G4-ligand with a terminal azide.^25^ SPAAC requires a constrained alkyne moiety, which was introduced by reacting the 5′-end primary amine of the oligonucleotide with (1*R*,8*S*,9*S*)-bicyclo[6.1.0]non-4-yn-9-ylmethyl *N*-succinimidyl carbonate (BCN-NHS ester) (Scheme 1b). This was followed by the azide–alkyne cycloaddition reaction with the G4-ligand containing a terminal azide, resulting in **GL-O5** (Figure 1c and Table S2). Compared with the CuAAC approach, the cyclooctane ring in SPAAC makes the linker slightly bulkier than the triazole alone and requires a two-step synthesis. However, this approach is applicable to a wider substrate scope, removes the need for copper, and results in equivalent overall yields (Table S3).

Finally, to further probe the importance of the length of the linker, both the amide coupling and SPAAC conjugation strategies were used to generate 12-carbon linker analogues, **GL-O4** and **GL-O6**, respectively (Figure 1c and Table S2).

### Impact of Different GL-O Linker Designs on G4 Binding and Stabilization

**Figure 2:**
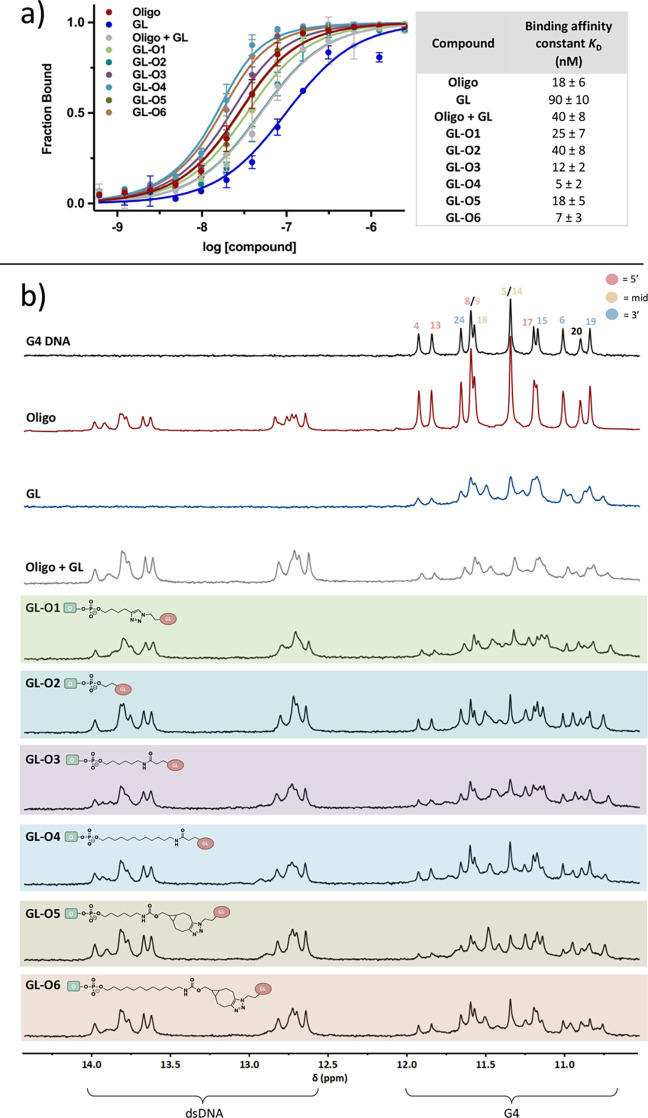
Impact of chemical composition and linker length on the ability of the GL-Os to bind G4 DNA. (a) Dose–response curves obtained using MST, and GL-Os were titrated to *c-MYC* Pu24T containing the complementary flanking sequence with a 5́-labeled fluorescent tag. Binding affinity constants (*K*_D_) values and error bars correspond to 3–4 independent measurements. (b) ^1^H NMR spectra of *c-MYC* Pu24T with complementary flanking sequence (90 μM) (black) in the presence of GL-Os **1–6**, oligo (red), or G4-ligand (GL) (blue), oligo and GL added together but not linked (gray), at 1:1 molar ratios. G4 imino signals appear between 10 and 12 ppm and double-stranded DNA signals appear between 12 and 14 ppm.

To assess the impact of the linker on the GL-O strategy, we targeted the mutated *c-MYC* G4 known as Pu24T, a parallel G4 structure commonly used for in vitro assessments. In our experiments, Pu24T is accompanied by a 15 nt flanking sequence at the 5′-end of the G4-forming sequence (Figure 1c and Table S1). From this point forward, G4 DNA refers to *c-MYC* Pu24T including the flanking sequence. By using this sequence, we first determined the binding affinity, the dissociation constants (*K*_D_), for the linker-modified GL-Os using MST with a fluorescently labeled G4 DNA. When *c-MYC* Pu24T was used as the target, all six GL-Os (**GL-O1** to **6**) displayed clear dose–response relationships and similar *K*_D_ values, ranging from 5 to 40 nM (Figure 2a). To validate these binding affinities, the endogenous *c-MYC* sequence known as Pu27, along with its adjacent 15 nt sequence from the NHEIII region of the MYC promoter, was also used to evaluate binding affinity (Figure S1 and Table S1). For this purpose, a set of corresponding GL-Os with the Pu27 complementary guide oligonucleotide sequence was also prepared (**GL-O7** to **12**, Figure S1a). The same trends were observed for *c-MYC* Pu27, indicating that these linker designs can be applicable to different targets (Figure S1b).

The binding affinity assay revealed that the GL-O with the shortest linker, **GL-O2**, has the highest *K*_D_ value (40 nM), significantly higher than the GL-Os containing longer linkers from the SPAAC and amide conjugation strategies with *K*_D_ values ranging between 5 and 18 nM (Figure 2a). This difference could partially be attributed to variations in the chemical compositions of the linkers. However, **GL-O3** and **GL-O5**, both with a 6-carbon linker and different linkage types, show similar *K*_D_ values. A slight difference in binding affinity is observed when comparing the GL-Os with different linker lengths within and between the SPAAC and amide conjugation strategies (amide: **GL-O3** and **GL-O4**, SPAAC: **GL-O5** and **GL-O6**) (Figure 2a).

For comparison, the oligonucleotide alone has a *K*_D_ of 18 nM, while the G4-ligand alone has a *K*_D_ of 90 nM. The combination of the oligonucleotide and G4-ligand, without being chemically linked, gives a *K*_D_ of 40 nM (Figure 2a). Overall, these results suggest that the binding affinities of the GL-Os are primarily determined by DNA/DNA duplex hybridization. This underscores the efficacy of the guide oligonucleotide in precisely directing the G4 ligand to its intended target. However, it is important to emphasize that the G4 ligand is essential for the ability of the GL-Os to stabilize G4 structures.

To enhance the resolution of the GL–O binding event, ^1^H NMR was employed. In ^1^H NMR, the signals from imino protons differ between G4 DNA and double-stranded DNA (dsDNA) (10–12 ppm for G4 DNA and 12–14 ppm for dsDNA), allowing for independent studies of the binding to the G4 structure and to the flanking sequence. Hence, ^1^H NMR spectra were recorded at 25 °C to study the binding of GL-Os 1–6 in 1:1 molar ratios with *c-MYC* Pu24T G4. The ^1^H NMR spectra of GL-Os 1–6 show that the dsDNA imino peaks remained consistent across all GL-Os, indicating successful hybridization (Figure 2b). Additionally, new G4 imino peaks emerged for GL-Os 1–6, similar to those observed for the G4-ligand alone, suggesting that the G4-ligand retains the same binding interactions with the G4 structure regardless of linker type and length.

**Figure 3:**
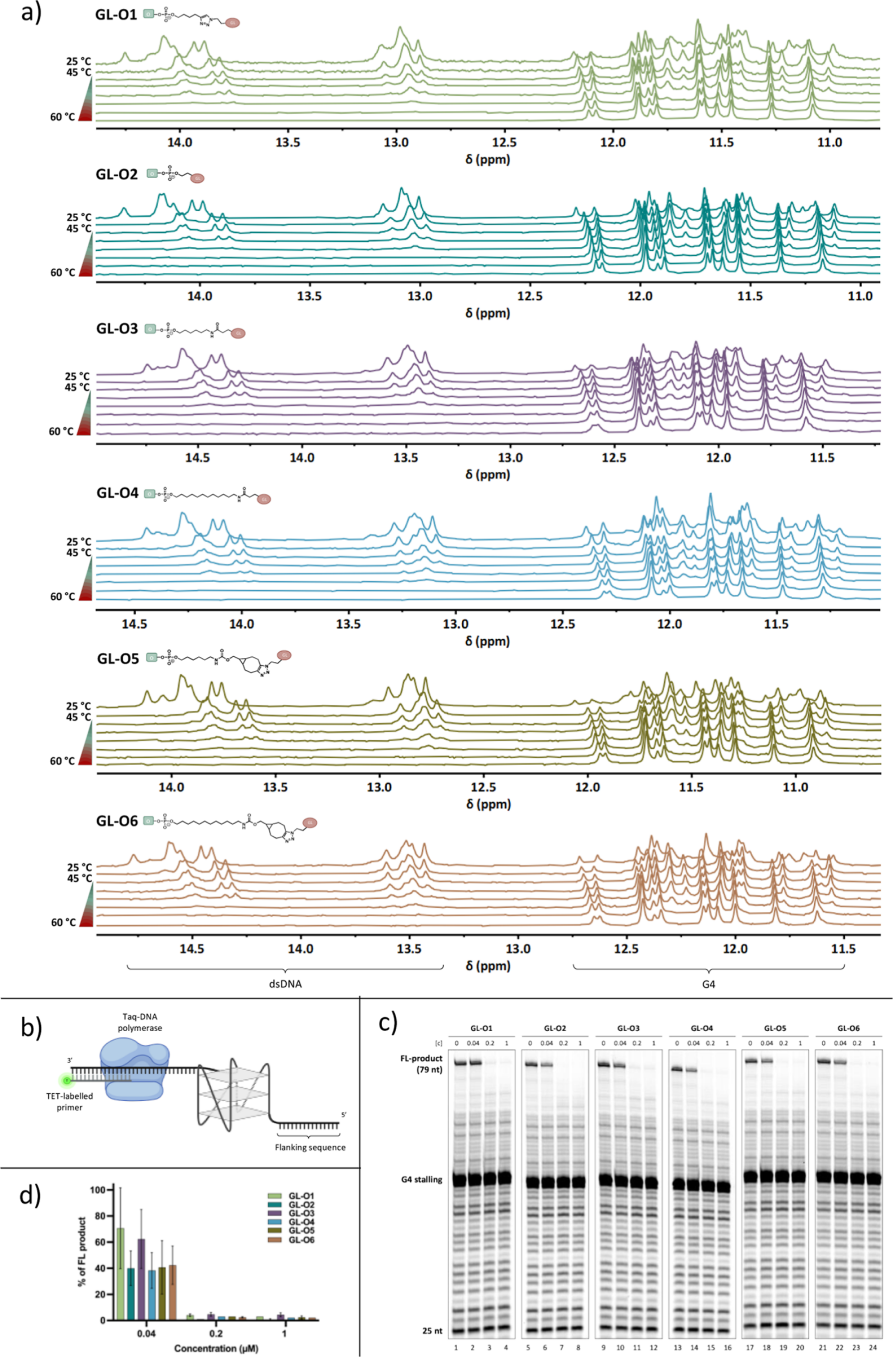
Impact of chemical composition and linker length of GL-Os on their ability to stabilize G4 DNA. (a) Thermal melting analysis using ^1^H NMR. Spectra were recorded of *c-MYC* Pu24T with complementary flanking sequence in the presence of GL-Os **1–6** (1:1 molar ratio) at 25–60 °C. G4 imino signals appear between 10 and 12 ppm and double-stranded DNA signals appear between 12 and 14 ppm. (b) Schematic representation of the Taq polymerase stop assay setup using a TET-labeled primer which is annealed to the G4-forming DNA template and extended by Taq Polymerase. (c) A representative gel from a Taq polymerase stop assay conducted in the presence of increasing concentration (0.04–1 μM) of **GL-O1** (lanes 1–4), **GL-O2** (lanes 5–8), **GL-O3** (lanes 9–12), **GL-O4** (lanes 13–16), **GL-O5** (lanes 17–20), and **GL-O6** (lanes 21–24). (d) Quantification of the Taq polymerase stop assay shown in c. The full-length (FL, 79 nt) DNA product is expressed as a % of the full-length band intensity that was observed in the control reaction containing only the G4 template. Data represent the mean ± standard deviation from three independent experiments.

To next investigate the ability of GL-Os 1–6 to stabilize the target G4 structure, we conducted thermal melting experiments between 25 and 60 °C with a 2.5 °C stepwise temperature increase using ^1^H NMR (Figure 3a). We have previously shown that dsDNA melts at a lower temperature compared to G4 DNA and that the GL-O modality can stabilize both the G4 DNA structure and the oligonucleotide hybridization to the sequence flanking the G4.^25^ Analysis of GL-Os 1–6 confirms this stabilization effect and reveals similar overall *T*_m_ profiles (Figure 3a). However, **GL-O2**, with the shortest linker, exhibited a *T*_m_ approximately 2.5 °C lower than the longer linker GL-Os **4–6**, which is in line with the binding affinity data from MST. The GL-Os with 6-carbon linkers, **GL-O3** (amide) and **GL-O5** (SPAAC), displayed differences in G4 stabilization, with the SPAAC-generated **GL-O5** demonstrating superior performance. This may be attributed to differences in chemical composition or, more likely, the longer overall linker length produced by the SPAAC method. Interestingly, the amide-based **GL-O3** exhibited a melting profile similar to that of the original CuAAC-generated **GL-O1**, both having similar linker lengths. These findings suggest that the linker length plays a more critical role than chemical composition in determining G4 stabilization efficiency.

A Taq polymerase stop assay was next utilized to examine if the GL-O-induced thermal stabilization of the G4 structure correlates with a DNA polymerase-based measurement of G4 stabilization. In this assay, Taq polymerase synthesizes DNA by copying a template strand containing the target G4 sequence and structure (Figure 3b). When the polymerase encounters the G4 structure, it stalls due to the difficulty of bypassing the G4. However, some DNA polymerase molecules can bypass G4 and continue synthesizing DNA to produce a full-length DNA product. GL-Os that bind and stabilize the G4 structure are expected to reduce the amount of the full-length DNA product. The experimental setup was designed to enable the DNA polymerase to initiate synthesis from the 25 nt TET-labeled primer, encountering the G4 structure at the 20th base (Figure 3b).

All GL-Os 1–6 demonstrated strong dose-dependent G4 stabilization, resulting in a potent reduction of the full-length DNA product (Figure 3c,d). At the lowest concentration (0.04 μM, approximately 1:1 molar ratio), no significant differences were observed among the linker-modified GL-Os, suggesting that, in this assay, the G4 stabilization is unaffected by changes in linker composition or length.

### Altering the Distance between the G4 and the Guide Oligonucleotide Binding Sequence of the GL-O

We have demonstrated that there is great flexibility in the linker composition while preserving both the binding affinity and stabilization of the target G4. However, the composition, foremost the length, may become important if the position of the guide oligonucleotide binding sequence adjacent to the target G4 needs to be modified. To investigate this, we varied the distance between target G4 and the guide oligonucleotide binding sequence in the G4 template.

The original GL-O strategy was designed with a three-nucleotide gap (TGA) between the G4 structure and the guide oligonucleotide recognition sequence (Berner et al. 2024).^25^ To explore the possibility to modify this nucleotide gap, we designed five additional G4 templates (Table 1): two shorter templates with one- or two-nucleotide gaps (**1nt**, **2nt**) and three longer templates with five-, seven-, or nine-nucleotide gaps (**5nt**, **7nt**, and **9nt**). The shorter templates were constructed by removing T or TG from the original TGA gap, while the longer gaps were designed by adding thymine residues to increase the distance between the G4 structure and the guide oligonucleotide binding sequence.

**Table 1:**
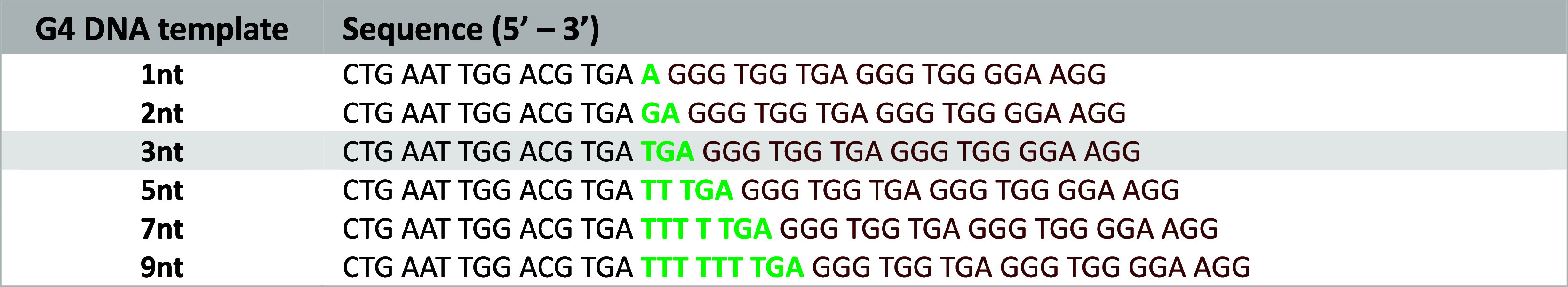
Alterations of the G4 DNA Template^a^

a Nucleotide gap in bold green and the G4-forming sequence in dark red.

To assess whether these target G4 sequences, with different distances between the G4 and the guide oligonucleotide binding sequence of the GL-Os, still form a G4 structure, we employed NMR. The ^1^H NMR experiments revealed that all new G4 templates displayed the same well-resolved *c-MYC* Pu24T G4 structure NMR signals (Figure S2), with one exception: the **1nt** template exhibited a slightly altered pattern of imino peaks (the imino proton signals for G-4 and G-13 merged, while those for G-5 and G-14 separated). However, the presence of clear imino proton signals in the same overall positions indicates that this template still forms a well-resolved G4 structure (Figure S2).

After determining that the different nucleotide gap sequences did not greatly impact the formation of the *c-MYC* Pu24T G4 structure, we next compared how the different nucleotide gaps affect the ability of GL-Os with different linker lengths to bind and stabilize the G4 using the same setup of biophysical and biochemical assays. Three GL-Os with different linker lengths were selected for these studies: **GL-O2** with the shortest linker length, **GL-O5** with an intermediate linker length, and **GL-O6** with the longest linker of all tested GL-Os (Figure 1c). First, MST was used to evaluate the binding affinity of GL-Os toward all six different G4 templates (Figure S3). This displayed no clear discrimination from any of the GL-Os toward the different nucleotide gaps in the G4 templates, which may be due to the significant influence of dsDNA hybridization on the overall *K*_D_ values of the GL-Os. However, the *K*_D_ values for **GL-O2** (SPOS-linker) were consistently higher than those for **GL-O5** and **GL-O6** (SPAAC-linkers), which is in line with previous MST studies.

**Figure 4:**
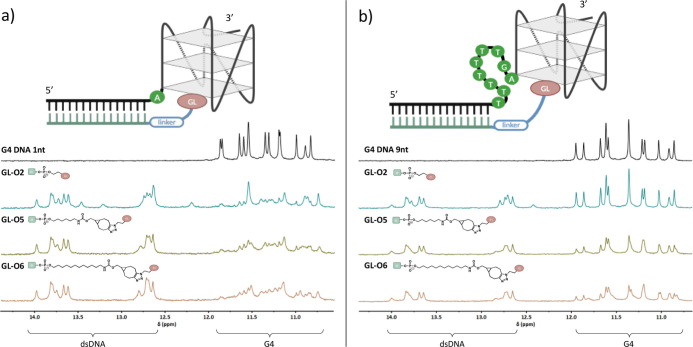
Impact of the distance between the G4 and the guide oligonucleotide binding sequence on the ability of the linker-modified GL-Os to bind the G4 structure using two templates **1nt** and **9nt**. (a) ^1^H NMR spectra of the **1nt** G4 DNA template in the presence of GL-Os **2**, **5**, and **6** in 1:1 molar ratios. (b) ^1^H NMR spectra of the **9nt** G4 DNA template in the presence of GL-Os **2**, **5**, and **6** in 1:1 molar ratios. G4 imino protons appear between 10 and 12 ppm and double-stranded DNA signals appear between 12 and 14 ppm.

We next proceeded to examine the interaction between GL-Os 2, 5, and 6 in a 1:1 molar ratio with the various G4 templates using ^1^H NMR. Overall, increasing the distance between the guide oligonucleotide binding sequence and the G4 reduces the GL-O’s ability to interact with the G4, with the extent of reduction varying depending on the linker length (Figures 4 and S4). The GL-O with the shortest linker, **GL-O2**, has a strong G4 interaction in the template with the shortest (**1nt**) nucleotide gap but shows a marked decrease in binding to the G4 structure as the recognition sequence is moved further away (Figure S4a). When the nucleotide gap is seven nucleotides or more, the interaction is completely lost. However, even though the G4-ligand of this GL-O is not able to bind the **7** or **9nt** G4 template, the guide oligonucleotide of the GL-O is still binding to form dsDNA. In contrast, the longer linkers in **GL-O5** and **GL-O6** result in strong G4 interactions with G4 templates having both shorter and longer nucleotide gaps (Figures 4 and S4b,c). When the G4 template contains a nine-nucleotide gap (**9nt**), a marked decrease in the ability of the G4-ligand in **GL-O5** and **GL-O6** to bind the G4 structure is observed although the guide oligonucleotide sequence still binds in a similar way (Figure 4b). These results corroborate that linker length is likely more important than chemical composition for efficient G4 binding and stabilization in this assay. Furthermore, this suggests that there is a critical limit where the linkers in GL-Os become too short for the G4-ligand to effectively reach the G4 as the distance between the G4 and the guide oligonucleotide binding sequence increases.

To investigate how the different nucleotide gaps between the G4 and the guide oligonucleotide binding sequence affect the ability of the GL-Os to stabilize the G4 structure, we measured thermal melting using ^1^H NMR. Adding the GL-Os to the different G4 templates (**1–9nt**) and recording ^1^H NMR spectra at increasing temperatures reveal notable differences in G4 stabilization between the different nucleotide gaps. Specifically, the *T*_m_ values of the GL-Os for the shorter nucleotide gaps (**1–3nt**) were similar, while a stepwise reduction in *T*_m_ was observed as the distance between the recognition sequence and the G4 increased (**5**, **7**, **9nt**) (Figures S5–S8).

**Figure 5:**
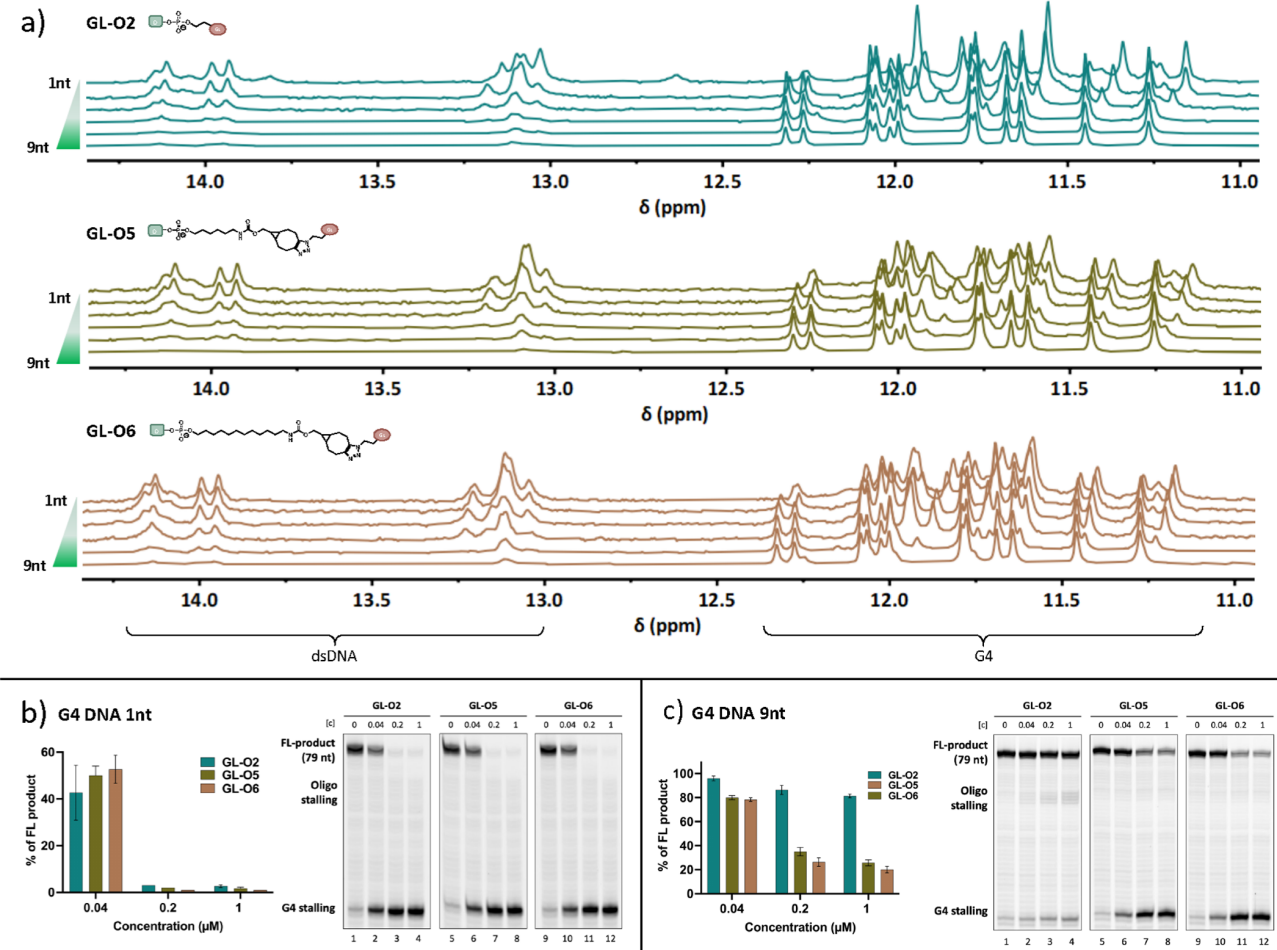
Impact of the distance between the G4 and the guide oligonucleotide binding sequence on the ability of GL-Os to stabilize the G4 structure. (a) Thermal melting analysis using ^1^H NMR recorded at 50 °C of GL-Os **2–4** in a 1:1 molar ratio with different G4 templates having modified distances between the G4 and the guide oligonucleotide binding sequence (**1**, **2**, **3**, **5**, **7**, **9nt** gaps). Full melting studies can be found in Figures S7–S10. Taq polymerase stop assay at 50 °C in the presence of increasing concentrations (0.04–1 μM) of **GL-O2** (lanes 1–4), **GL-O5** (lanes 5–8), and **GL-O6** (lanes 9–12) using (b) **1nt** and (c) **9nt** at G4 DNA templates. Lower part of the gels is cropped. Quantification of the full-length (FL) DNA product or G4 stalling is expressed as a % of the full-length or G4 stalling band intensity obtained in the control reaction containing only the G4 template. Mean and standard deviation of three individual experiments are shown.

At 50 °C, the G4-ligand in the GL-O with the shortest linker, **GL-O2**, was efficiently stabilizing the G4 DNA templates with the shorter (**1–3nt**) nucleotide gaps. However, this stabilization decreased significantly as the nucleotide gaps increased, showing only weak stabilization for the **5nt** gap and no binding or stabilization for the **7nt** and **9nt** G4 DNA templates (Figure 5a). Increasing the linker length, as in **GL-O5** and **GL-O6**, also resulted in efficient stabilization with the shorter nucleotide gaps, despite the risk for these longer and bulkier SPAAC-generated linkers to induce steric hindrance. Compared to the shorter linker in **GL-O2**, the longer linkers in **GL-O5** and **GL-O6** also maintain G4 stabilization with the **5nt** and **7nt** nucleotide gap sequences at this temperature (Figure 5a). Analyzing these linker and nucleotide gap variations across all temperatures (25, 45, 50, 55, and 60 °C) confirmed these results and revealed a clear correlation between stabilization of the G4 DNA structure and the oligonucleotide hybridization to the sequence flanking the G4 (Figures S6–S8). These findings underscore the importance of this combined effect and confirm that incorporating a longer linker between the G4-ligand and the guide oligonucleotide in the GL-O facilitates the design of an effective GL-O, even when the distance between the target G4 structure and the guide oligonucleotide binding sequence is increased.

To assess whether these differences in thermal stabilization are reflected in DNA polymerase-based measurements of G4 stabilization, we employed the Taq-polymerase stop assay. Using G4 templates with the shortest and longest (**1nt** and **9nt**) gaps, we measured the amount of the full-length product synthesized in the presence of GL-Os 2, 5, and 6. These assays revealed dose-dependent G4 stabilization, with all GL-Os showing a strong reduction in the full-length DNA product with no apparent differences in G4 stabilization between the GL-Os for the **1nt** and the **9nt** gap G4 templates, similarly to the original **3nt** gap template (Figures S9 and S10).

To challenge the GL-Os further, we increased the incubation temperature from 37 to 50 °C in the Taq-polymerase stop assay. This increased incubation temperature did not alter the results for GL-Os 2, 5, and 6 when the **1nt** G4 template was used (Figure 5b). However, a clear difference emerged when the **9nt** gap template was used in the assay with an incubation temperature of 50 °C: **GL-O2**, with the shorter SPOS-generated linker, showed noticeably weaker G4 stabilization compared with **GL-O5** and **GL-O6** with longer SPAAC-generated linkers. This difference was evident from the quantification of both the full-length DNA product and the G4 stalling bands (Figure 5c). Notably, the guide oligonucleotide alone and **GL-O2**, which has the shorter SPOS-generated linker, induced polymerase stalling downstream of the G4. This stalling likely occurs when the polymerase encounters the dsDNA formed through hybridization of the guide oligonucleotide with its complementary binding sequence. This phenomenon was only observed with the **9nt** gap template and was independent of incubation temperature (Figures 5c and S10c). In contrast, the more efficient **GL-O5** and **GL-O6** exhibited minimal polymerase stalling downstream of G4, suggesting that this stalling is linked to weaker stabilization of the G4 structure.

Taken together, these findings suggest that when the system is challenged by increased temperatures, the combined action of GL-O with both efficient oligonucleotide and G4-ligand binding becomes essential for efficient G4 stabilization. This highlights the critical role of the linker in the design of efficient GL-Os. An extended SPAAC-generated linker, such as in **GL-O6**, improves binding affinity and stabilization across a wider range of G4 targets and does not appear to impose any major steric hindrance, even when the guide oligonucleotide binding sequence is in close proximity to the G4 target. This flexibility is particularly valuable in scenarios where multiple G4 structures or isomers form within a single sequence or when the lack of a unique DNA sequence near the G4 necessitates designing the guide to anneal further away for improved selectivity.

### Computational Modeling of G4 in Complex with Linker-Modified GL-Os

**Figure 6:**
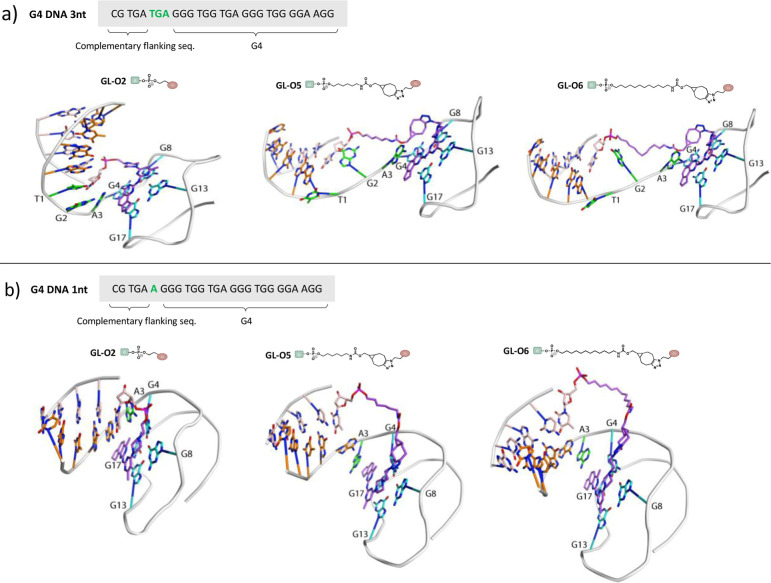
Computational modeling of G4 DNA templates (a) **3nt** and (b) **1nt** of *c-MYC* Pu24T with a 5 nt flanking sequence in complex with GL-Os **2**, **5**, and **6**.

To visualize and further understand how the linker-modified GL-Os bind and interact with the G4 structure, we modeled the *c-MYC* Pu24T G4 (PDB: 2MGN)^35^ in complex with GL-Os **2**, **5**, and **6** (Figure 6). To reduce the overall size of the complex, we used a shorter 5 nt flanking sequence, as this modification will not change the position of the linker or G4-ligand (Table S1). The models were prepared using MD simulations to investigate the conformational landscape of the G4-oligonucleotide, molecular docking of selected ligands, followed by modeling of the linker, as outlined in Figure S11a.

All of the GL-Os **2**, **5**, and **6** were successfully modeled to the G4 DNA without displaying any conflicting interactions or strain, thus corroborating the experimental data showing that GL-Os **2**, **5**, and **6** are all able to bind the G4 structure regardless of linker type and length (Figure 6a). These models also highlight the flexibility of the system, where the G4 flanking sequence can adopt multiple conformations, thereby allowing all GL-Os to be positioned such that the G4 ligand can interact with the top G-tetrad of the G4 structure. However, **GL-O2**, with the shortest linker, displayed difficulty in locating a conformational state of the G4 that enabled modeling of the linker, suggesting that the short two-carbon linker makes it harder to find a favorable G4 interaction.

When the distance between the G4 and the guide oligonucleotide binding sequence was shortened to a 1 nt gap, GL-Os **2**, **5**, and **6** were still able to find suitable G4 interactions (Figure 6b). These models also reinforce the high adaptability of the system, as even **GL-O6**, with the longest bulky SPAAC linker, was able to find a favorable binding pose. Increasing the distance between the G4 and the guide oligonucleotide binding sequence to a 9 nt gap prevented modeling of the GL-Os **2**, **5**, and **6** to any of the generated conformational states of the G4. The flexibility of the system is likely difficult to recapitulate in the computational model, which may explain this discrepancy with the results from the other assays. Nevertheless, this reinforces that when the guide oligonucleotide binding sequence is positioned further away from the G4, there is an increased risk of reduced GL–O binding and stabilization of the target G4, unless a corresponding extension of the linker length is introduced.

Overall, the computational and experimental data align, showing that a long and flexible linker can facilitate G4 binding across a broader range of G4 targets with varying distances between the guide oligonucleotide binding sequence and the G4. Moreover, the models suggest that flexibility in the linker is preferable; a more rigid linker, such as in **GL-O2**, is less likely to adopt the required conformations, leading to reduced or failed G4 binding.

## Conclusions

The GL-O strategy was recently presented as a promising approach to enable stabilization of individual G4 structures using a guide oligonucleotide conjugated to a G4-ligand (GL-Os).^25^ Central to this approach is the linker between the G4 ligand and the guide DNA oligonucleotide. In this work, we systematically developed various conjugation methods, each offering distinctive advantages. Compared to the previously used CuAAC, the copper-free solution-phase methods developed herein, including strain-promoted click chemistry and amide couplings, offer versatile options for a wide variety of substrates by using standard functional handles on both the ligand and the guide oligonucleotide. Additionally, SPOS offers an automated and efficient approach for GL-O synthesis, allowing the integration of diverse G4 ligands as phosphoramidites. Combined, these developments enhance the versatility of the GL-O platform by facilitating diverse conjugation strategies and linker configurations, enabling the integration of various G4-ligands and guide oligonucleotides.

In addition to evaluating the different conjugation methods, we also tested GL-Os with varying linker lengths for their ability to bind and stabilize G4s using biophysical, biochemical, and computational methods. This suggested that the linker length is likely more important than the chemical composition for efficient G4 binding and stabilization. GL-Os with extended SPAAC-linkers proved particularly advantageous, enabling efficient G4 binding and stabilization across a broad range of targets regardless of whether the guide oligonucleotide binding sequence was positioned near or far from the G4. Furthermore, extended linkers were particularly important under conditions of thermal stress, where the combined binding of both the guide oligonucleotide and the G4-ligand from GL-O was crucial for effective G4 stabilization. Importantly, the extended GL-O linkers do not appear to introduce significant steric hindrance, even when the guide oligonucleotide binding sequence is positioned in close proximity to the G4 target. The possibility to use extended linkers is especially beneficial in systems where multiple G4 structures or isomers can form or when modifications to the guide oligonucleotide binding sequence are required to target genomic flanking regions not directly adjacent to the G4.

Overall, the insight gained from this study on conjugation strategies, linker length designs, and computational models can now be applied to fine-tune the linkers for targeting various G4 structures that naturally occur within the genome in a cellular context. The ability to fine-tune the GL-O strategy will be instrumental in advancing its development. This refined approach offers a unique method for investigating the biological functions of specific individual G4 structures within living cells while also holding potential as a novel therapeutic modality acting at the DNA level to target these critical genomic elements.

## Data Availability

Detailed synthetic procedures and conjugation methods, experimental data for the assays, and a more detailed description of the computational modeling are provided in the Supporting Information.

## Supporting Information Available

Detailed experimental information for compound synthesis, GL-O conjugations, and in vitro assays; NMR spectra of compounds; chromatograms of GL-O conjugates; and computational modeling (PDF)
