# Supplementary material for: Linker Design Principles for the Precision Targeting of Oncogenic G-Quadruplex DNA with G4-Ligand-Conjugated Oligonucleotides
Source: Bioconjug Chem. 2025 Mar 20;36(4):724–36. doi: 10.1021/acs.bioconjchem.5c00008 (PMC12006964; doi:10.1021/acs.bioconjchem.5c00008)
Supplement: Supplementary file 1 — bc5c00008_si_001.pdf [file bc5c00008_si_001.pdf]

Electronic Supplementary Information

Linker Design Principles for the Precision Targeting of Oncogenic G-quadruplex DNA with G4-Ligand Conjugated Oligonucleotides

Alva Abrahamsson[a], Andreas Berner[b], Justyna Golebiewska-Pikula[a,c], Namrata Chaudhari[b], Emelie Keskitalo[a], Cecilia Lindgren[a], Marcin K. Chmielewski[c,d], Sjoerd Wanrooij[b]*, Erik Chorell[a]*

Table of content

Table S1. G4 DNA templates 3
Table S2.......... 4
Figure S1. 4
Figure S2. 5
Figure S3. 5
Figure S4. 6
Figure S5. 7
Figure S6. 8
Figure S7. 9
Figure S8. 10
Figure S9. 11
Figure S10 11
General Experimental 12
Synthesis 12
NMR spectra 17
Chromatograms of the GL-O conjugates 22
Table S3.......... 25
In vitro assays 26
Computational modelling 26
Table S4.......... 27
Figure S11. 28
Figure S12. 29
Figure S13. 30
Figure S14. 31
References 32

Table S1. G4 DNA templates

Used in	G4 DNA	Sequence (5´-3´)
NMR assay	*c-MYC* Pu24T **3nt**	CTG AAT TGG ACG TGA TGA GGG TGG TGA GGG TGG GGA AGG
MST assay	*c-MYC* Pu24T **3nt**	[Cy5]-CTG AAT TGG ACG TGA TGA GGG TGG TGA GGG TGG GGA AGG
Computational modelling	*c-MYC* Pu24T **3nt**	CG TGA TGA GGG TGG TGA GGG TGG GGA AGG
MST assay	*c-MYC* Pu27	GAG GGG CGC TTA TGG GGA GGG TGG GGA GGG TGG GGA AGG
NMR assay	*c-MYC* Pu24T **1nt**	CTG AAT TGG ACG TGA A GGG TGG TGA GGG TGG GGA AGG
MST assay	*c-MYC* Pu24T **1nt**	[Cy5]-CTG AAT TGG ACG TGA A GGG TGG TGA GGG TGG GGA AGG
Computational modelling	*c-MYC* Pu24T **1nt**	CG TGA A GGG TGG TGA GGG TGG GGA AGG
NMR assay	*c-MYC* Pu24T **2nt**	CTG AAT TGG ACG TGA GA GGG TGG TGA GGG TGG GGA AGG
MST assay	*c-MYC* Pu24T **2nt**	[Cy5]-CTG AAT TGG ACG TGA GA GGG TGG TGA GGG TGG GGA AGG
NMR assay	*c-MYC* Pu24T **5nt**	CTG AAT TGG ACG TGA TT TGA GGG TGG TGA GGG TGG GGA AGG
MST assay	*c-MYC* Pu24T **5nt**	[Cy5]-CTG AAT TGG ACG TGA TT TGA GGG TGG TGA GGG TGG GGA AGG
NMR assay	*c-MYC* Pu24T **7nt**	CTG AAT TGG ACG TGA TTT T TGA GGG TGG TGA GGG TGG GGA AGG
MST assay	*c-MYC* Pu24T **7nt**	[Cy5]-CTG AAT TGG ACG TGA TTT T TGA GGG TGG TGA GGG TGG GGA AGG
NMR assay	*c-MYC* Pu24T **9nt**	CTG AAT TGG ACG TGA TTT TTT TGA GGG TGG TGA GGG TGG GGA AGG
MST assay	*c-MYC* Pu24T **9nt**	[Cy5]-CTG AAT TGG ACG TGA TTT TTT TGA GGG TGG TGA GGG TGG GGA AGG
Computational modelling	*c-MYC* Pu24T **9nt**	CG TGA TTT TTT TGA GGG TGG TGA GGG TGG GGA AGG
Polymerase Stop assay	*c-MYC* Pu24T **3nt**	CTG AAT TGG ACG TGA A GGG TGG TGA GGG TGG GGA AGG CAC GTG AGT TGA GTG GAG TTG GAA GTA GGC ATA CCC CTA T
Polymerase Stop assay	*c-MYC* Pu24T **1nt**	CTG AAT TGG ACG TGA TGA GGG TGG TGA GGG TGG GGA AGG CAC GTG AGT TGA GTG GAG TTG GAA GTA GGC ATA CCC CTA T
Polymerase Stop assay	*c-MYC* Pu24T **9nt**	CTG AAT TGG ACG TGA TTT TTT TGA GGG TGG TGA GGG TGG GGA AGG CAC GTG AGT TGA GTG GAG TTG GAA GTA GGC ATA CCC CTA T
Polymerase Stop assay	*c-MYC* Pu24T **90nt** circle	GAG CGT TAC AAC TTG CTG AAT TGG ACG TGA TGA GGG TGG TGA GGG TGG GGA AGG CAC TGA GTA TGG AGT TGG AAG TAG GCC CTA ATC TGA
Polymerase Stop assay	*c-MYC* Pu24T **120nt circle**	GAG CGT TAC AAC TTG CTG AAT TGG ACG TGA TGA GGG TGG TGA GGG TGG GGA AGG CAC TGA GTA TGG AGT TGG AAG TAG GCG AGG TAA AGC CAG TCA CCC AGT GTC TAG GCC CTA ATC TGA
Polymerase Stop assay	15nt TET primer	[TET]-ATA GGG GTA TGC CTA CTT CCA ACT C
Polymerase Stop assay	25nt TET primer	[TET]-GCC TAC TTC CAA CTC
Polymerase Stop assay	15nt ddC blocker	CAA GTT GTA ACG CT[ddC]
Polymerase Stop assay	Circularization oligo	CAA GTT GTA ACG CTC TCA GAT TAG GGC CTA

G4 forming sequence (red), nucleotide gap (green), primer site (blue), circularization binding site (underlined)

Table S2. GL-O conjugates synthesized in this study

Conjugate name	Conjugation method	Oligo 5´modification	Sequence (5´-3´)
**GL-O1**	CuAAC	Hexynyl	TCA CGT CCA ATT CAG (for *c-MYC* Pu24T)
**GL-O2**	SPOS	No linker	
**GL-O3**	Amide coupling	Amine-C6	
**GL-O4**	Amide coupling	Amine-C12	
**GL-O5**	SPAAC	Amine-C6	
**GL-O6**	SPAAC	Amine-C12	
**GL-O7**	CuAAC	Hexynyl	CCA TAA GCG CCC CTC (for *c-MYC* Pu27)
**GL-O8**	SPOS	No linker	
**GL-O9**	Amide coupling	Amine-C6	
**GL-O10**	Amide coupling	Amine-C12	
**GL-O11**	SPAAC	Amine-C6	
**GL-O12**	SPAAC	Amine-C12	

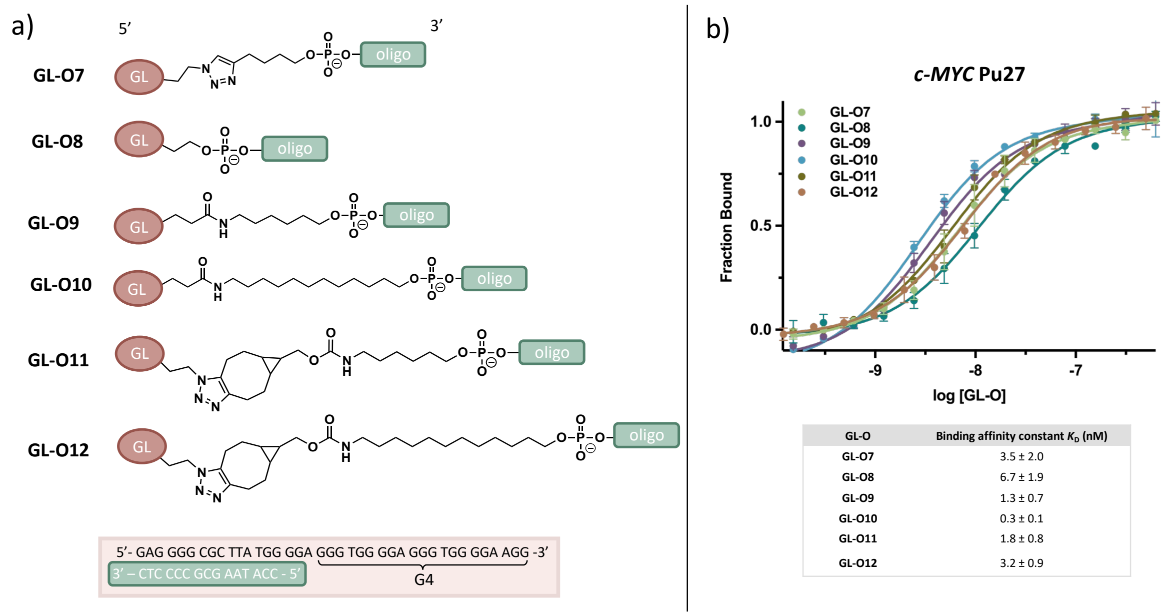

Figure S1. Validation of the modified linker GL-Os binding affinity to the G4 target, in this case c-MYC Pu27. a) The GL-Os made for this target. b) Dose-response curves from MST analysis where GL-Os **7-12** were titrated onto *c-MYC* Pu27 with putative flanking sequence (see sequence in Table S1) and a 5′ labelled fluorescent tag. Binding affinity constants (K_D) and error bars correspond to two independent measurements**.**

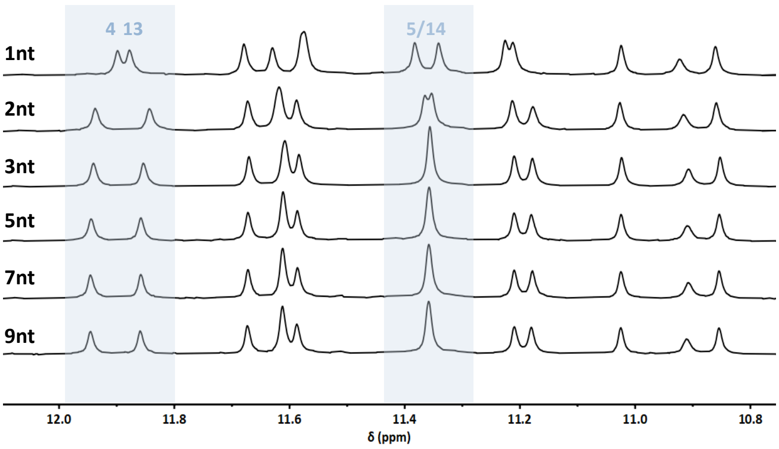

Figure S2. Varying the distance between G4 and the complementary flanking sequence (see templates in Table S1). ^{1}H NMR spectra of the different G4 DNA templates (90 µM) in absence of GL-O. Highlighting the guanine imino protons G-4, G-13, G-5 and G-14.

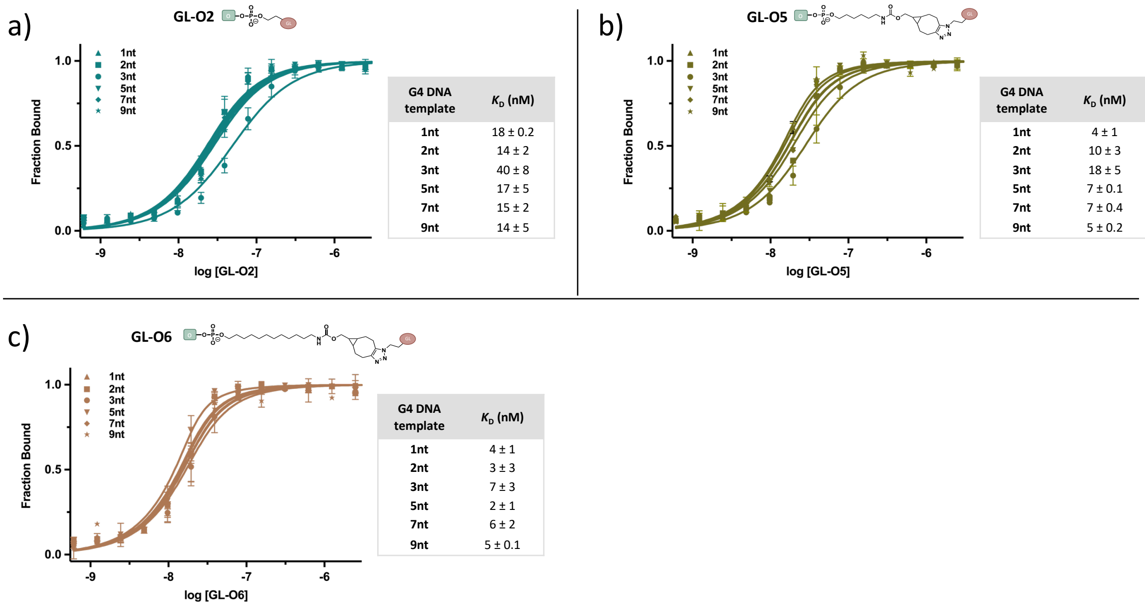

a)

G4 DNA template	K_D (nM)
1nt	18 ± 0.2
2nt	14 ± 2
3nt	40 ± 8
5nt	17 ± 5
7nt	15 ± 2
9nt	14 ± 5

b)

G4 DNA template	K_D (nM)
1nt	4 ± 1
2nt	10 ± 3
3nt	18 ± 5
5nt	7 ± 0.1
7nt	7 ± 0.4
9nt	5 ± 0.2

c)

G4 DNA template	K_D (nM)
1nt	4 ± 1
2nt	3 ± 3
3nt	7 ± 3
5nt	2 ± 1
7nt	6 ± 2
9nt	5 ± 0.1

Figure S3. Dose-response curves from MST analysis where GL-Os a) **2** b) **5** and c) **6** were titrated onto the different G4 DNA templates containing a 5' labelled fluorescent tag (see sequence in Table S1). Binding affinity constants (K_D) and error bars correspond to two independent measurements**.**

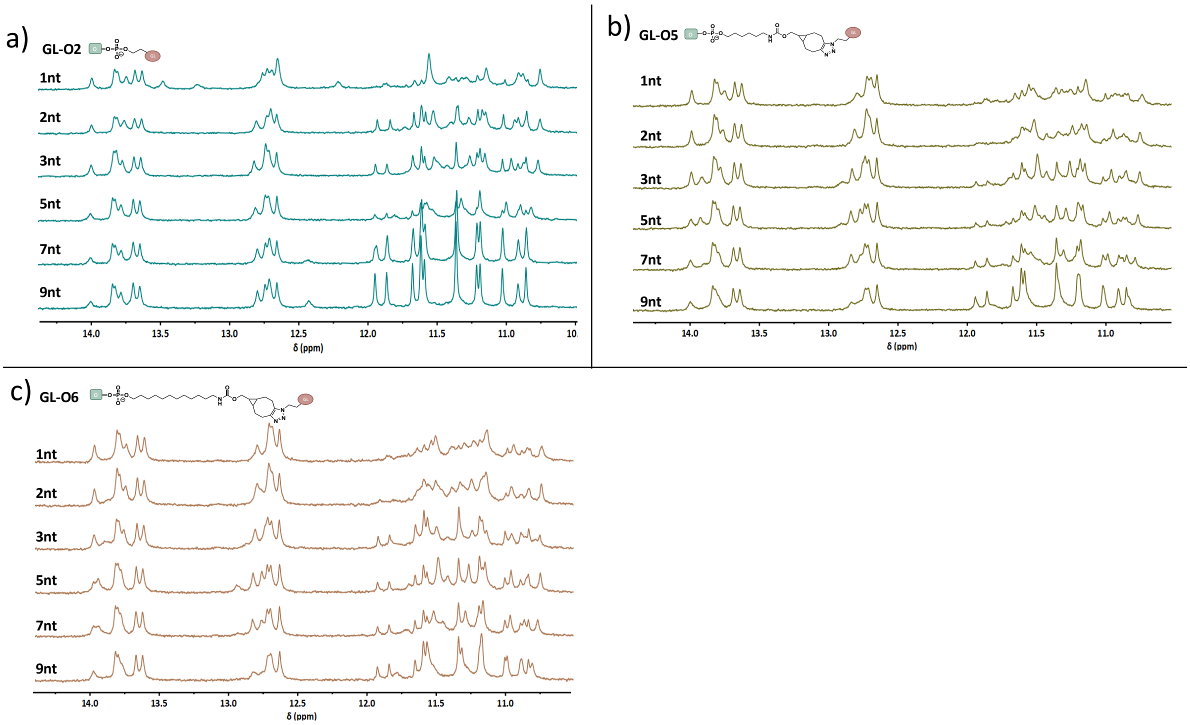

Figure S4. Varying the distance between G4 DNA and the complementary flanking sequence and the impact on G4 binding of different linker length GL-Os. ^{1}H NMR spectra of *c-MYC* Pu24T with complementary flanking sequence (90 µM) in presence a) **GL-O2**, b) **GL-O5**, c) **GL-O6**. G4 imino signals appear between 10-12 ppm and double-stranded DNA signals appear between 12-14 ppm.

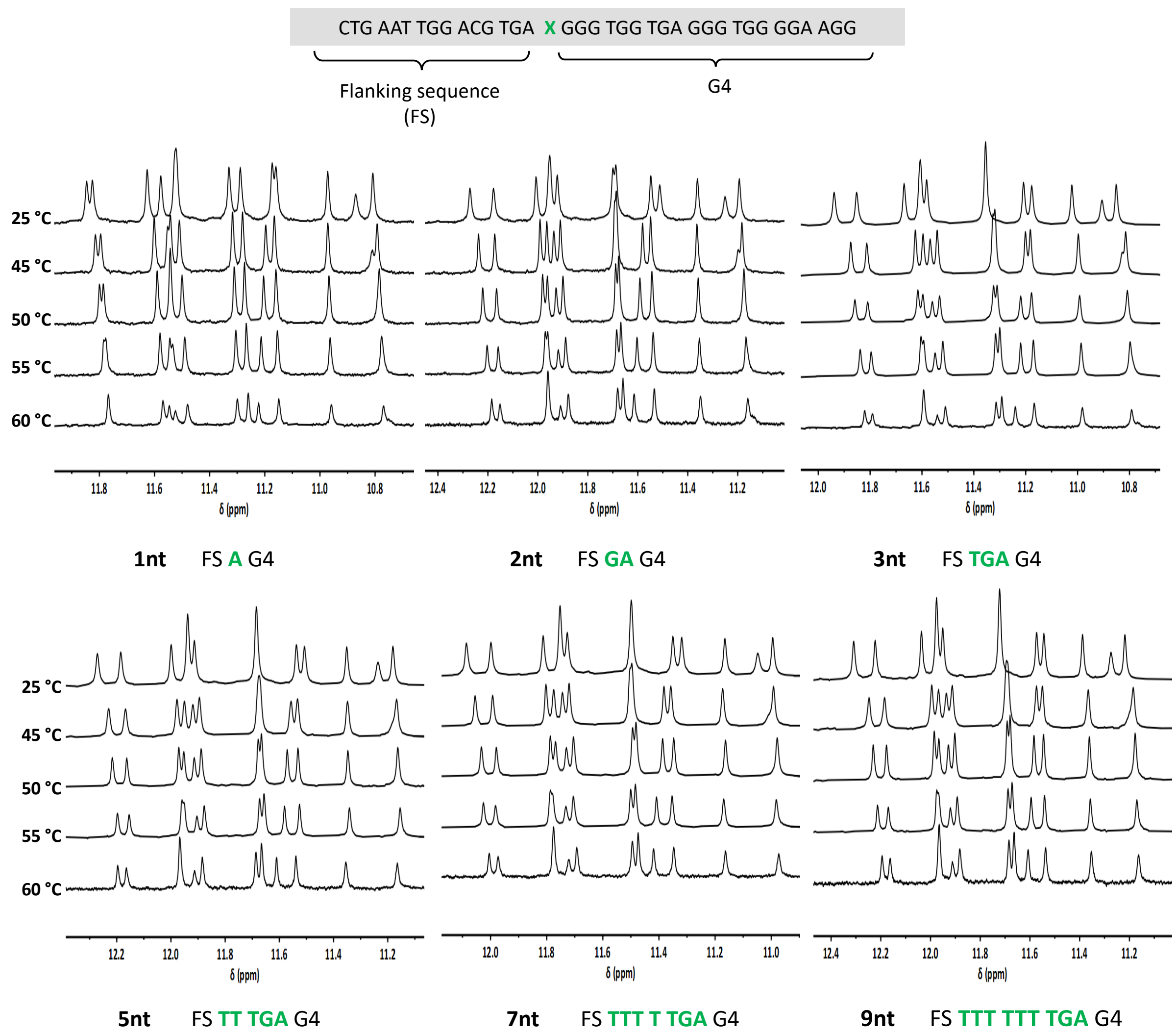

Figure S5. Thermal melting analysis using ^{1}H NMR. Spectra were recorded of the different distance G4 DNA templates (see sequences in Table S1) without adding GL-O. G4 imino signals appear between 10-12 ppm.

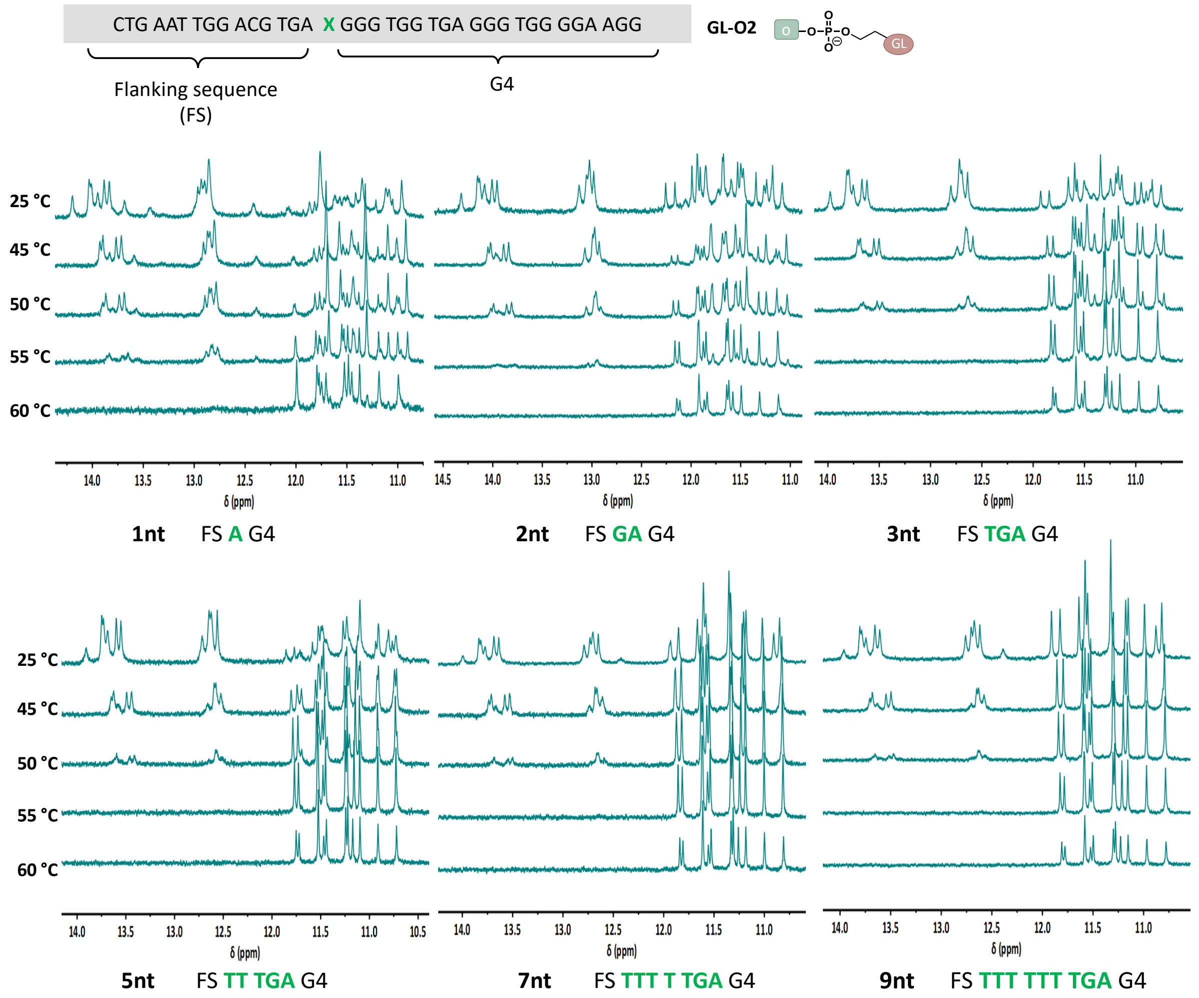

Figure S6. Thermal melting analysis using ^{1}H NMR. Spectra were recorded of the different distance G4 DNA templates (see sequences in Table S1) with added **GL-O2** (1:1 molar ratio). G4 imino signals appear between 10-12 ppm and double-stranded DNA between 12-14 ppm.

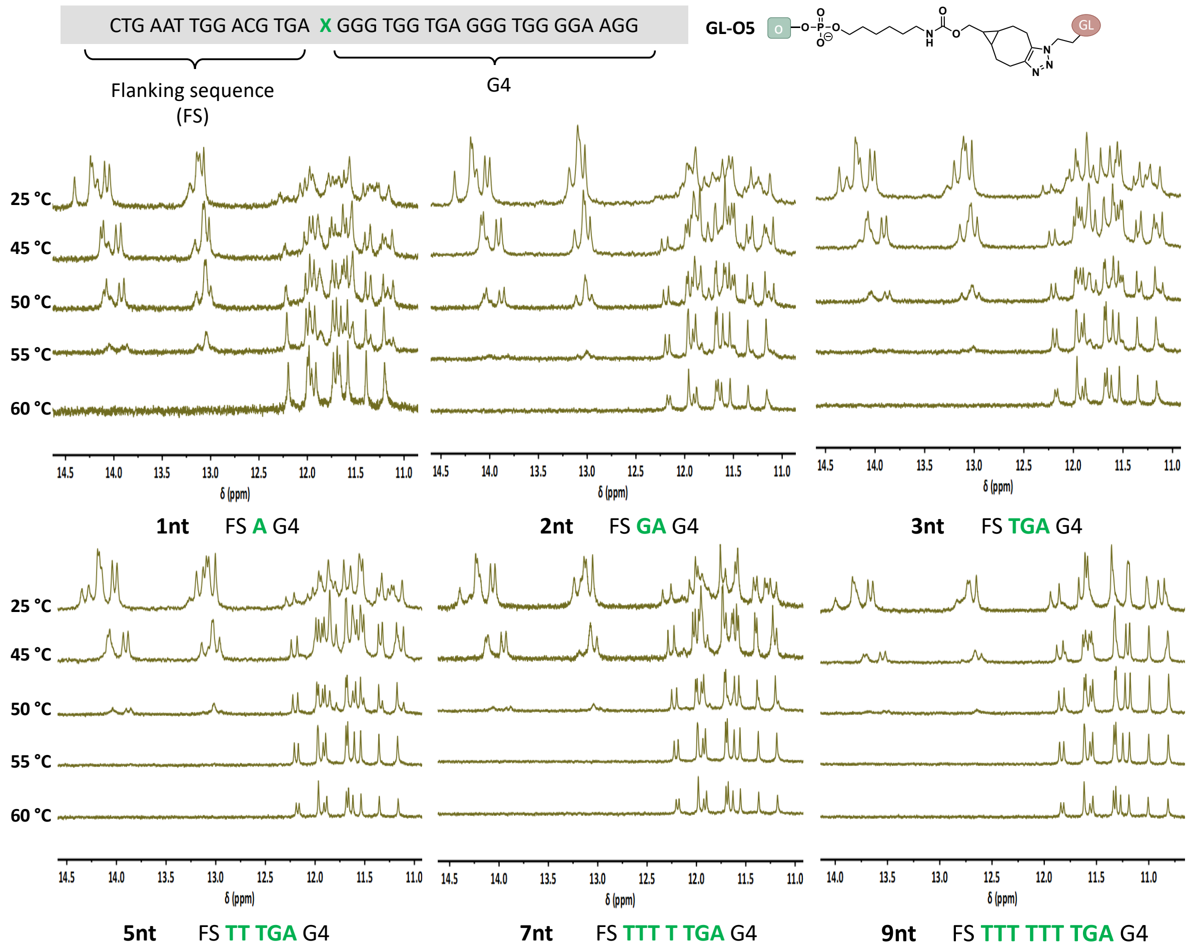

Figure S7. Thermal melting analysis using ^{1}H NMR. Spectra were recorded of the different distance G4 DNA templates (see sequences in Table S1) with added **GL-O5** (1:1 molar ratio). G4 imino signals appear between 10-12 ppm and double-stranded DNA between 12-14 ppm.

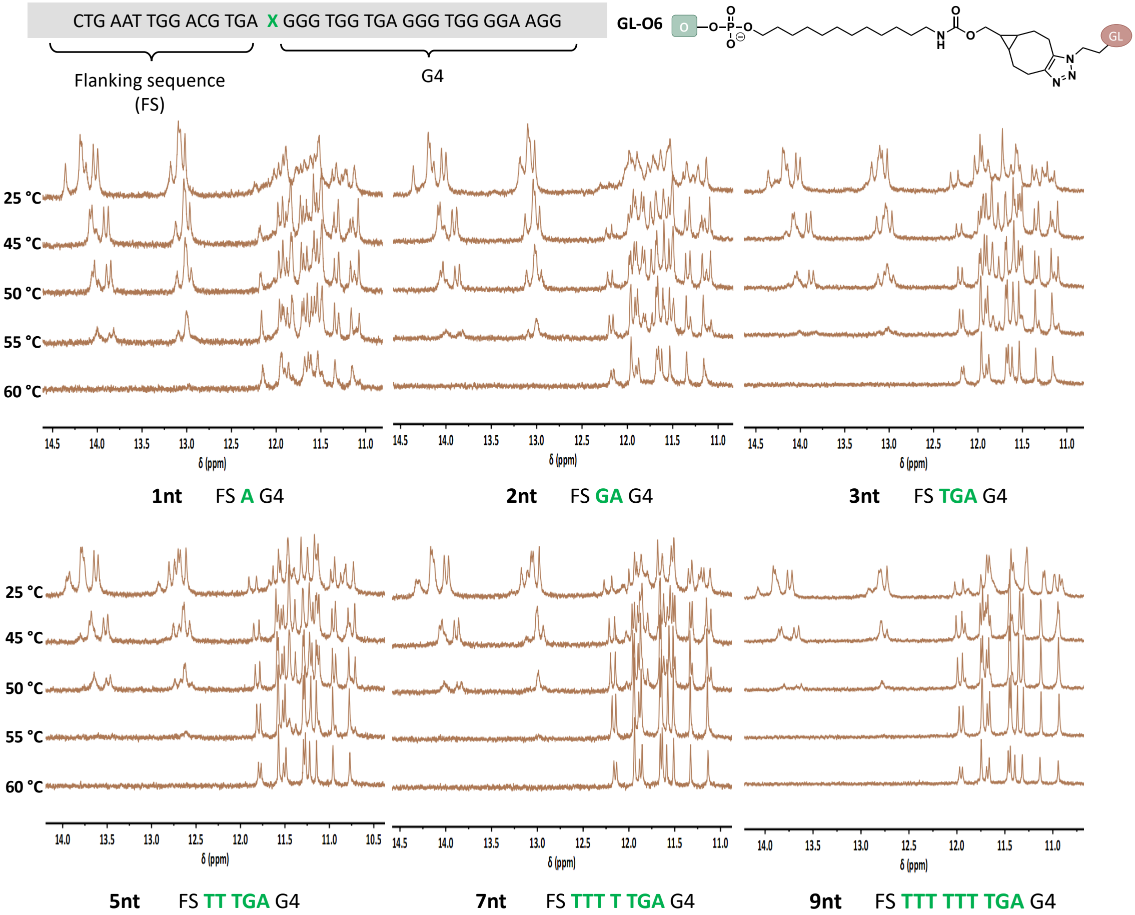

Figure S8. Thermal melting analysis using ^{1}H NMR. Spectra were recorded of the different distance G4 DNA templates (see sequences in Table S1) with added **GL-O6** (1:1 molar ratio). G4 imino signals appear between 10-12 ppm and double-stranded DNA between 12-14 ppm.

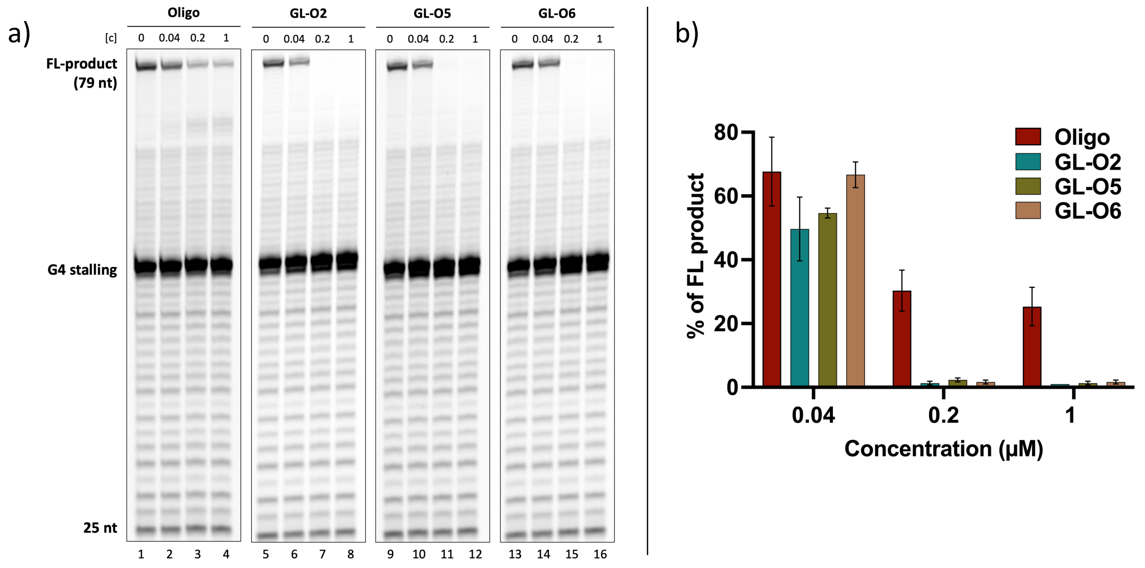

Figure S9. Taq Polymerase Stop assay of **1nt** G4 DNA template at 37°C, in presence of increasing concentration (0.04-1 µM) of oligo (lanes 1-4), **GL-O2** (lanes 5-8) and **GL-O5** (lanes 9-12), **GL-O5** (lanes 13-16).

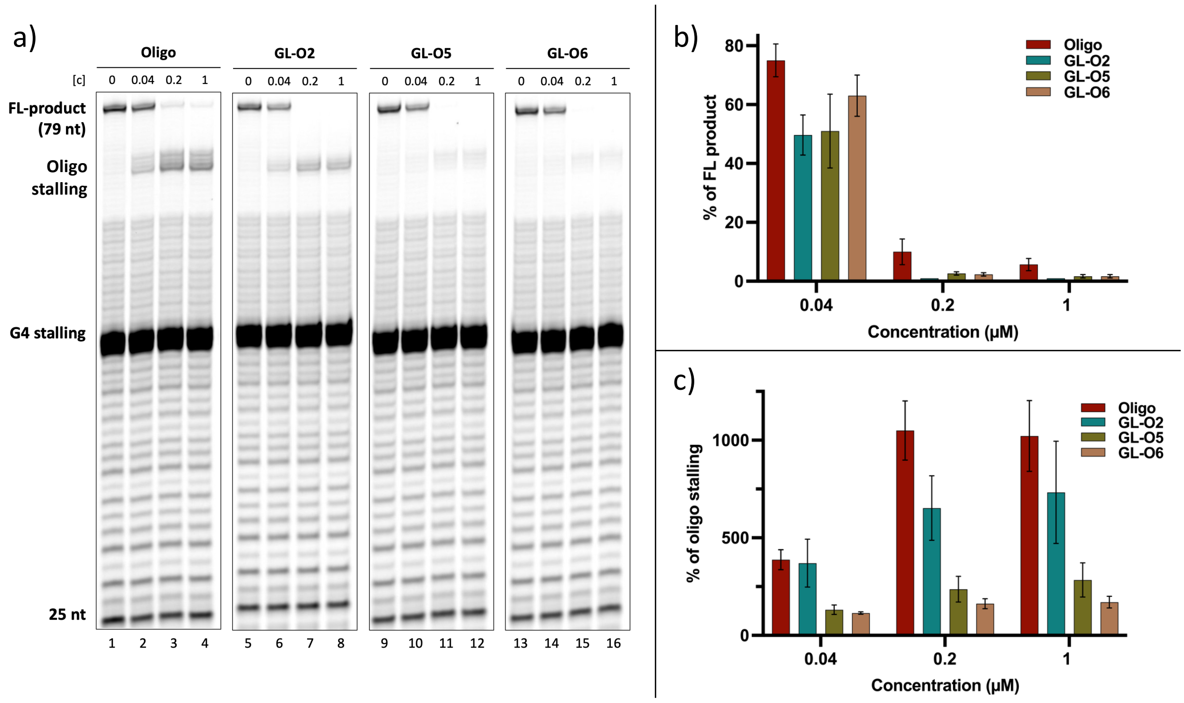

Figure S10. Taq Polymerase Stop assay of **9nt** G4 DNA template at 37°C. a) **9nt** G4 DNA template with increasing concentration (0.04-1 µM) of oligo (lanes 1-4), **GL-O2** (lanes 5-8), **GL-O5** (lanes 9-12) and **GL-O6** (lanes 13-16). b) Quantification of the full-length (FL) product is expressed in % of full-length band intensity that was obtained in the control reaction only containing the G4 template. c) Quantification of oligo stalling in % of oligo stalling. Mean and standard deviation of three individual experiments is shown.

General Experimental

Safety statement. The work conducted during this project adhered to standard laboratory safety protocols and procedures. No unexpected safety situations or hazards were encountered, and the materials and methods employed did not require any precautions beyond routine laboratory safety measures. Proper handling, storage, and disposal practices were followed throughout the project to ensure a safe working environment.

For compound synthesis: All reagents and solvents were purchased from commercial suppliers unless stated otherwise. TLC was performed on aluminum backed silica gel plates (median pore size 60 Å, florescent indicator 254 nm) and detected with UV light. Flash column chromatography was performed using silica gel with an average particle diameter of 50 µm (range 40−65 µm, pore diameter 53 Å), or aluminum oxide 150 basic (63-200 µm), eluents are given in brackets. ^{1}H and ^{13}C NMR spectra for characterization were recorded on a Bruker 400 MHz spectrometer at 298 K or on a Bruker 600 MHz spectrometer at 298 K and calibrated by using the residual peak of the solvents as the internal standard ($CDCl_3$: δ H = 7.26 ppm; δ C = 77.16 ppm. DMSO-d_6: δ H = 2.50 ppm; δ C = 39.50 ppm). LC-MS was conducted on an Agilent 6150 Series Quadrupole LC/MS system. HRMS was performed by using an Agilent 1290 binary LC System connected to an Agilent 6230 Accurate-Mass TOF LC/MS (ESI^+); calibrated with Agilent G1969-85001 ESTOF Reference Mix containing ammonium trifluoroacetate, purine and hexakis (1H, 1H, 3H tetrafluoropropoxy) phosphazine in 90:10 ACN/H_2O.

For oligonucleotide conjugation: All reagents and solvents including N,N-diisopropylethylamine (DIPEA), ethylenediamine tetraacetate (EDTA), triethylamine (TEA), acetic acid, acetonitrile (ACN, HPLC grade), methanol and dimethyl sulfoxide (DMSO), were purchased from *Merck Sigma Aldrich*, and 1,1,1,3,3,3-hexafluoro-2-propanol (HFP) from *Fluorochem*. The oligonucleotides were purchased from IDT (Integrated DNA Technologies, Sweden) and Eurofines Genomics (Germany). Water used for reactions and buffers were in Milli-Q purity. Reverse phase (RP) HPLC was carried out on a Hitachi HPLC system using Clarity 5 µm Oligo RP LC, fully porous Organo-silica C18 (250 x 10 mm) semi-preparative column (Phenomenex) with 2 mL/min flow rate with the method: 0-1 min 95% A, 1-15 min 95-40% A, 15-16 min 40-5% A, 16-19 min 5% A, 19-20 min 5-95% A, 20-24 min 95% A, using detection at 260 nm at room temperature. The buffers used for RP-HPLC were as follows: (A) 50 mM triethylammonium acetate (TEAA), pH ~6.5; (B) 100% acetonitrile. TEAA (1 M) buffer was prepared by dropwise addition of glacial acetic acid (57 mL) to a cooled and stirred mixture of triethylamine (139 mL) in water (800 mL). The pH was adjusted with diluted acetic acid to ~7 and the volume was adjusted to 1 L with water. Characterization was done using a HRMS electrospray time-of-flight (ES-TOF) Agilent instrument. Oligonucleotides were eluted using an aqueous mixture of HFP and TEA with increasing gradient of methanol.

Synthesis

Synthesis of the G4-ligand containing a terminal azide has been described in Berner. A, et al. *JACS*. 2024.[1]

Copper-mediated azide-alkyne cycloaddition:

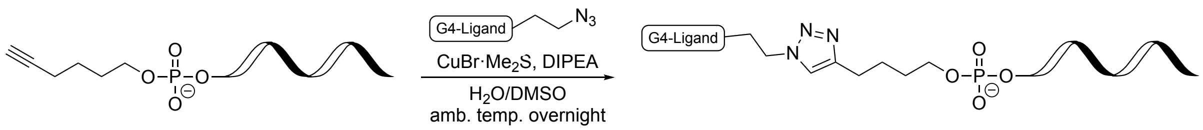

Oligo (50 nmol) was dissolved to 1 mM in Milli-Q water. After that, reagents were added in the following order: G4-ligand (25 µL, 5 equiv. 10 mM stock in ACN/DMSO 7:3), 0.1 M DIPEA (aq) solution (2.5 µL) and 67 mM $CuBr{\cdot}Me_2S$ solution in DMSO (7.5 µL). The reaction mixture was vortexed and let agitate at ambient temperature overnight. The reaction was quenched with 0.5 mM EDTA solution (25 µL) and filtered before purification. The conjugate was purified by RP-HPLC with 2 mL/min flowrate and a linear gradient of 5 to 60% solvent B (100% ACN). Solvent A was 50 mM TEAA buffer pH 7.0. The conjugate was confirmed with HRMS (ESI^-).

Strain-promoted azide-alkyne cycloaddition:

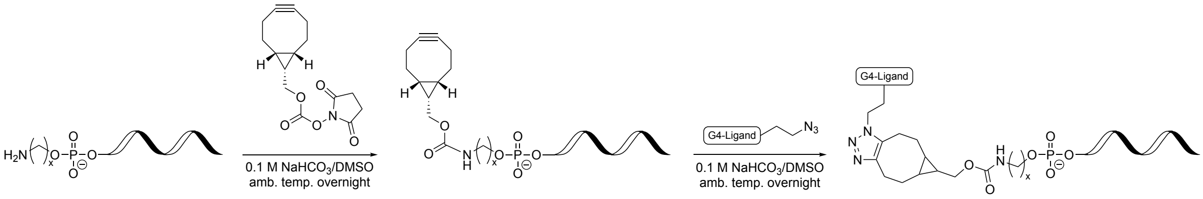

Oligo (Na^+ salt, 50 nmol) was dissolved in 0.1 M $NaHCO_3$ (aq) to a 1 mM solution. A freshly prepared solution of (1R,8S,9s)-bicyclo[6.1.0]non-4-yn-9-ylmethyl N-succinimidyl carbonate (BCN-NHS ester) in DMSO (5 µL, 20 equiv., 200 mM) was added to the oligo solution. The reaction mixture was vortexed and let agitate in ambient temperature overnight. The oligo was next precipitated using 10% v/v of 3 M NaOAc and 4x volume of EtOH, incubated in the freezer for 30 min, followed by centrifugation for 15 min and removing the supernatant. The precipitation was repeated one time followed by a wash of the pellet using only EtOH. The pellet was dried using a gentle N_2 gas flow and then resuspended in 0.1 M $NaHCO_3$ (aq) (50 µL).

G4-ligand containing a terminal azide (12.5 µL, 2.5 equiv. 10 mM in DMSO) was added to the dissolved oligo. The resulting solution was vortexed and let agitate for overnight. The reaction mixture was then filtered and purified by RP-HPLC. Purification was done by applying a linear gradient of 5 to 60% solvent B (100% ACN). Solvent A was 50 mM TEAA buffer pH 7. The flowrate was 2 mL/min flowrate. The conjugation product was confirmed with HRMS (ESI^-).

Synthesis of compound 2:

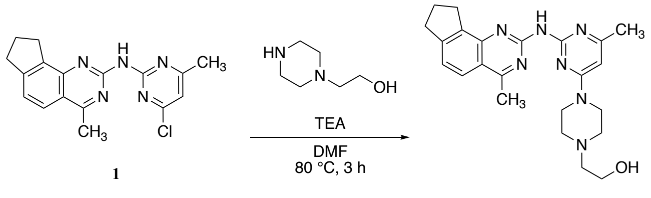

Precursor compound **1** has previously been synthesized and described in Bhuma, N. et al. *Eur J Med Chem*. 2023.[2] A microwave vial was charged with compound **1** (100 mg, 0.31 mmol) and 1-(2-hyrdoxyethyl)piperazine (60 mg, 0.46 mmol) in dry DMF (2 mL), followed by the addition of triethylamine (0.11 mL, 0.77 mmol). The reaction mixture was heated at 80 °C for 3 h. After cooling down, DMF was evaporated under reduced pressure and the crude was purified by SiO_2 column chromatography (eluent: 8% MeOH and 0.4% NH_3 in DCM). Product **2** was afforded as a yellow solid (112 mg, 87%). ^1H NMR (400 MHz, Chloroform-*d*) δ 7.76 (d, *J* = 8.3 Hz, 1H), 7.27 (d, *J* = 8.3 Hz, 1H), 6.09 (s, 1H), 3.80 (t, 4H), 3.68 (t, *J* = 5.3 Hz, 2H), 3.28 (t, *J* = 7.5 Hz, 2H), 3.08 (t, *J* = 7.4 Hz, 2H), 2.80 (s, 3H), 2.61 (t, *J* = 4.8 Hz, 6H), 2.34 (s, 3H), 2.20 (p, 2H). ^{13}C NMR (151 MHz, DMSO-d_6) δ 168.98, 162.35, 157.48, 154.68, 149.64, 147.40, 138.72, 124.10, 121.12, 119.06, 95.13, 60.25, 58.50, 52.94, 48.61, 43.50, 33.73, 30.03, 24.08, 23.37, 21.48. MS (ES mass): *m/z* 420.3 (*M+1*).

Synthesis of compound 3:

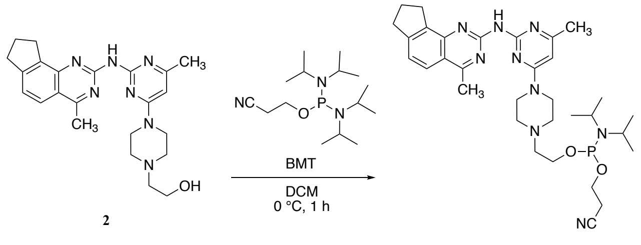

Compound **2** (41 mg, 0.1 mmol) was diluted in DCM to obtain a 0.2 M solution. Then 2-cyanoethyl *N,N,N′,N′*-tetraisopropylphosphorodiamidite (30 mg, 0.1 mmol, 0.033 mL) was added while cooling using an ice bath. After that, 5-(benzylthio)-1*H*-tetrazole (BMT) (7.7 mg, 0.04 mmol) was added and the reaction was stirred while cooling using an ice bath for 1 h. The progress of the reaction was monitored by ^{31}P NMR where the chemical shift of the starting material was 122 ppm, and the product 148 ppm. After all starting material was consumed, an excess of DIPEA (10 equiv., 126 mg, 1 mmol, 0.17 mL) was added and the resulting mixture was evaporated to dryness. The final product was purified using SiO_2 column chromatography (eluent: a mixture of DCM: *n*-hexane: TEA (6:3:1, v/v/v). The phosphoramidite **3** was obtained as a solid and due to instability it was immediately used for conjugation with the oligonucleotide without further characterization. ^{31}P NMR (162 MHz, H_2PO_4 as external standard) δ 148.00, -0.03.

Solid-phase oligonucleotide synthesis:

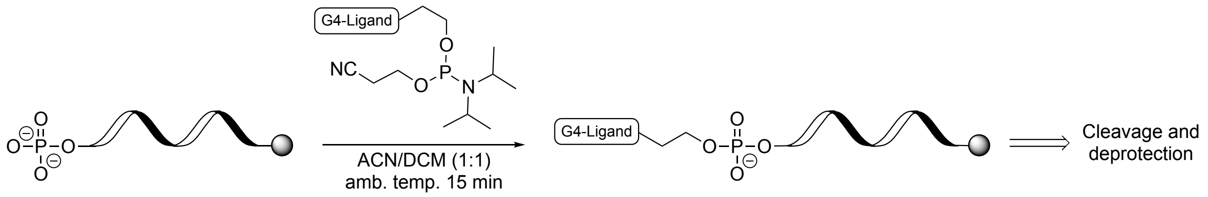

GL-Os were synthesized on a K&A oligonucleotides synthesizer (1 μmol scale) using dN(-) CPG (500 Å, ChemGenes) solid support. Fully protected DNA phosphoramidites were incorporated using standard oligonucleotide synthesis conditions, i.e., 3% dichloroacetic acid in DCM for deblocking, 0.25 M 4,5-dicyanoimidazole (DCI) in ACN as activator, a mixture of equal volumes of solution A (20% *N*-methylimidazole in ACN) and solution B (30% lutidine, 20% acetic anhydride, 50% ACN) for capping and 0.05 M iodine in 90% pyridine (aq) for oxidation. DNA phosphoramidites were dissolved in dry ACN (0.1 M) and injected over 1 min, see Table 4. G4-ligand phosphoramidite **3** was dissolved in ACN/DCM (1:1) to a 0.15 M solution and activated using 0.25 M 5-benzylmercaptotetrazole (BMT) in ACN, the mixture was allowed to react with the CPG support for 15 min, see Table 4.

After synthesis was completed, the oligonucleotide backbone along with nucleobase protecting groups was deprotected and cleaved from the solid support using 30% ammonia (aq) for 24 h at room temperature. Ammonia was next evaporated using a Speedvac, followed by purification on RP-HPLC

using a gradient of 0 to 100% solvent B (60% 0.1 M TEAA buffer containing 40% (v/v) ACN). Solvent A was 0.1 M triethylammonium acetate buffer pH 7, with 1% ACN. The conjugate product was confirmed by HRMS (ESI$^-$).

Synthesis of compound 4:

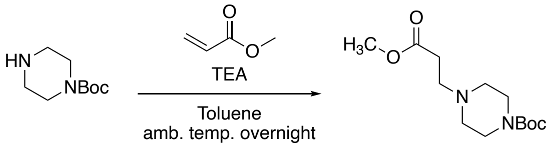

4

An RBF (25 mL) was charged with 1-boc-piperazine (200 mg, 1.07 mmol) and dissolved in DCM (2 mL), followed by trimethylamine (37 µL, 0.27 mmol) and methyl acrylate (0.11 mL, 1.18 mmol). The reaction was left stirring overnight at ambient temperature. The reaction mixture was next co-evaporated twice with toluene, to yield compound **4** as a light-yellow crude (233 mg, 80%). ^{1}H NMR (400 MHz, Chloroform-*d*) δ 3.68 (s, 3H), 3.41 (t, *J* = 5.1 Hz, 4H), 2.70 (t, *J* = 7.1 Hz, 2H), 2.51 (t, *J* = 7.3 Hz, 2H), 2.40 (t, *J* = 5.1 Hz, 4H), 1.45 (s, 9H).

Synthesis of compound 5:

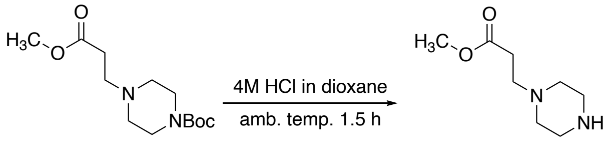

4 5

4M HCl in dioxane (3 mL) was added to the RBF containing compound **4** (233 mg, 0.85 mmol). The reaction mixture was stirred at ambient temperature for 1.5 h followed by co-evaporation with DCM, resulting in a fine white powder. The powder was redissolved in water and made basic with saturated $NaHCO_3$. The aqueous layer was extracted using ethyl acetate (3x25 ml), washed with brine and dried over sodium sulphate. The organic phase was filtered and concentrated under reduced pressure, to give compound **5** (47.4 mg, 32%). ^{1}H NMR (400 MHz, Chloroform-*d*) δ 3.68 (s, 3H), 2.90 (t, 4H), 2.69 (t, *J* = 7.2 Hz, 2H), 2.51 (t, *J* = 7.2, 2H), 2.45 (t, 4H).

Synthesis of compound 6:

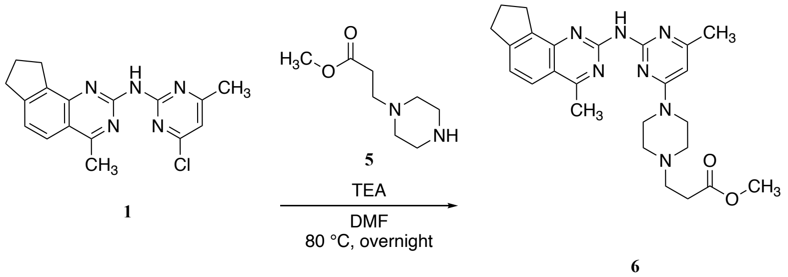

1

6

Compound **5** was dissolved in DMF (1.85 mL) and transferred into a microwave vial, followed by compound **1** (60 mg, 0.18 mmol) and triethylamine (128 mL, 0.92 mmol). The vial was sealed and the reaction mixture was heated at 80 °C and left stirring for 18 h. After cooling down, the DMF was co-

evaporated with toluene. The product was purified using basic alumina column chromatography (0.2-1% MeOH in DCM). Compound **6** was isolated as a yellow crude (41 mg, 48%). ^{1}H NMR (400 MHz, Chloroform-*d*) δ 7.78 (d, *J* = 8.3 Hz, 1H), 7.28 (d, *J* = 8.2 Hz, 1H), 6.02 (s, 1H), 3.78 (t, 4H), 3.71 (s, 3H), 3.32 (t, *J* = 7.4 Hz, 2H), 3.10 (t, *J* = 7.3 Hz, 2H), 2.82 (s, 3H), 2.77 (t, *J* = 7.3 Hz, 2H), 2.56 (t, *J* = 5.3 Hz, 6H), 2.33 (s, 3H), 2.22 (p, *J* = 7.6 Hz, 2H).

Synthesis of compound 7:

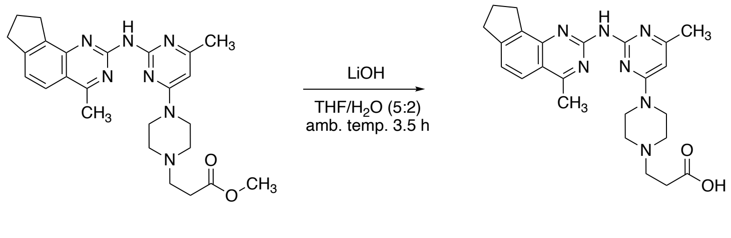

6 7

Compound **6** (41 mg, 0.09 mmol) was dissolved in THF (1.3 mL) and 0.15 M LiOH in THF/water (5:2) (0.5 mL). The reaction mixture was left stirring at ambient temperature for 3.5 h followed by removal of the THF under reduced pressure. The pH of the resulting mixture was adjusted to 7 using 1M HCl before being extracted using chloroform/isopropanol (3:1), resulting in compound **7** (10 mg, 25%). ^{1}H NMR (400 MHz, Methanol-d_4) δ 8.04 (d, *J* = 8.3 Hz, 1H), 7.54 (d, *J* = 8.4 Hz, 1H), 6.69 (s, 1H), 3.92 (t, 4H), 3.36 (t, *J* = 7.6 Hz, 2H), 3.21 (t, *J* = 7.6 Hz, 2H), 2.93 (s, 3H), 2.80 (t, *J* = 7.3 Hz, 2H), 2.67 (t, *J* = 6.1 Hz, 4H), 2.58 (s, 3H), 2.46 (t, *J* = 7.3 Hz, 2H), 2.35 (p, *J* = 7.6 Hz, 2H). ^{13}C NMR (151 MHz, DMSO-d_6) δ 173.47, 169.15, 162.32, 154.76, 149.83, 147.45, 138.81, 124.28, 121.29, 119.18, 95.30, 69.79, 53.23, 52.18, 43.43, 33.77, 31.69, 30.07, 24.20, 23.40, 21.57. MS (ES mass): *m/z* 448.2 (*M+1*)

Conjugation through amide coupling:

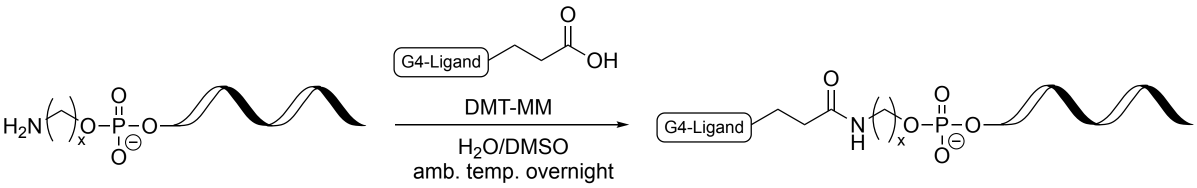

A 15 mM solution of 4-(4,6-dimethoxy-1,3,5-triazin-2-yl)-4-methyl-morpholinium chloride (DMT-MM) in DMSO was freshly prepared and 50 µl (750 nmol, 7.5 equiv.) was added to an Eppendorf tube. 50 µl (500 nmol, 5 equiv.) of a 10 mM solution of carboxylic acid G4-ligand **7** in DMSO and was added to the same Eppendorf tube. The reaction mixture was vortexed and left to react for 15 minutes while shaking. 100 µl of a 1 mM oligonucleotide solution (100 nmol, 1 equiv., in 0.1 M $NaHCO_3$ (aq)) was added to the reaction mixture. The reaction mixture was vortexed before it was left to react for 24 to 48 h on a shaker at room temperature. The reaction mixture was filtered before purified by RP-HPLC using a with a linear gradient of 5 to 95% of solvent B (100 % ACN) in solvent A (50 mM TEAA buffer, pH 7) using a flowrate of 2mL/min. The conjugate was confirmed with HRMS (ESI^-).

NMR spectra

Compound 2

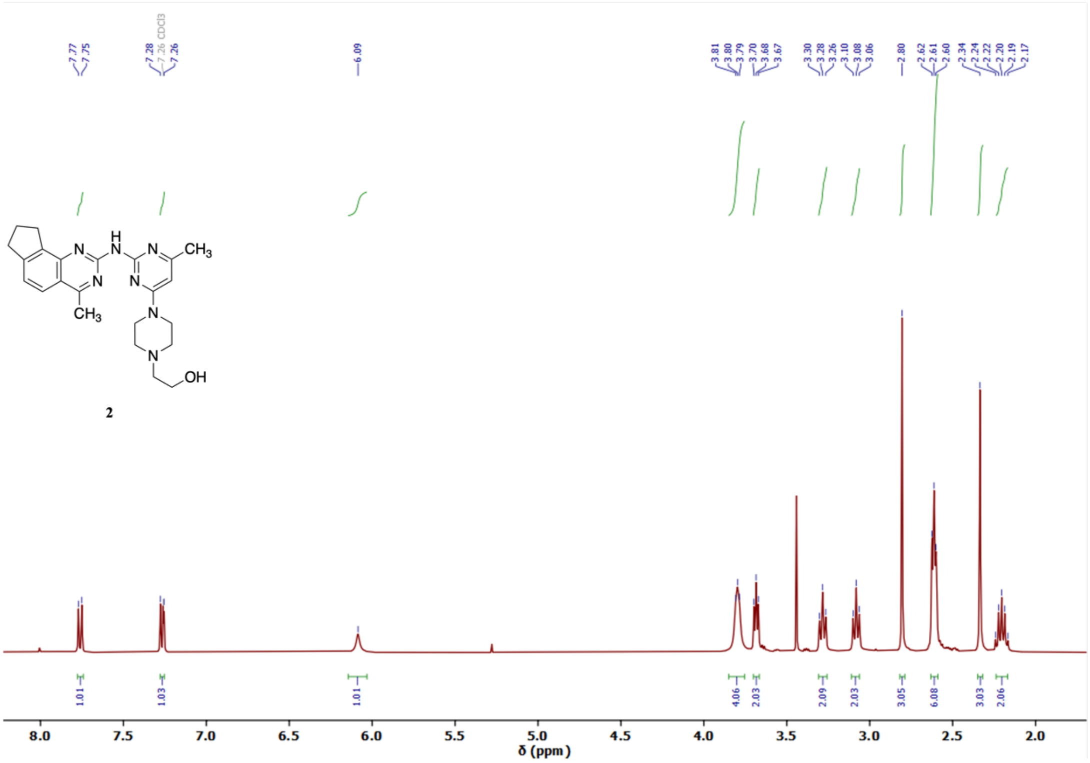

^{1}H NMR spectrum of **2** in chloroform-*d* measured at 400 MHz.
^{13}C NMR spectrum of **2** in DMSO-d_6 measured at 151 MHz.

Compound 3

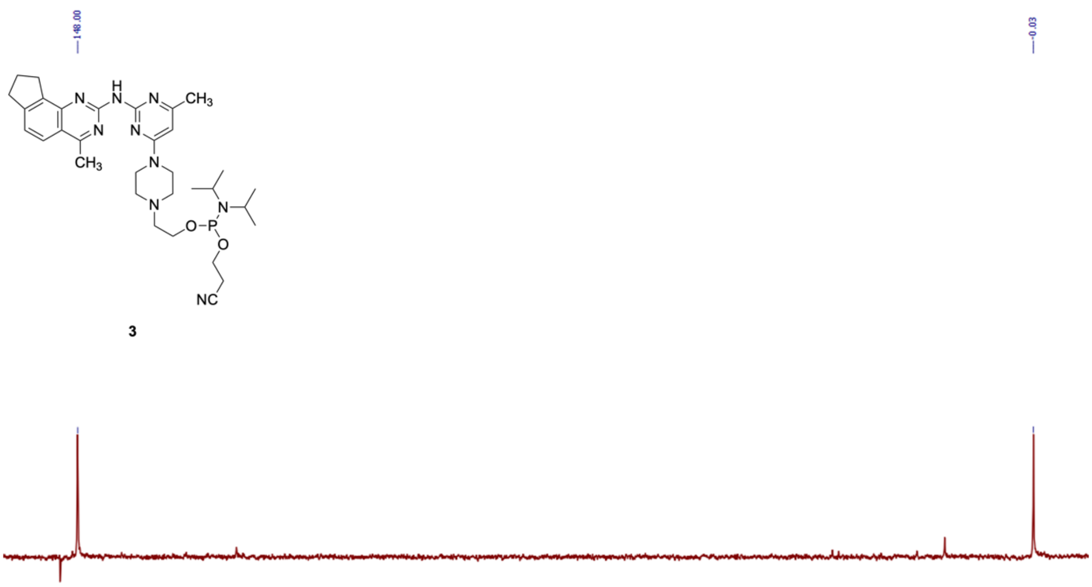

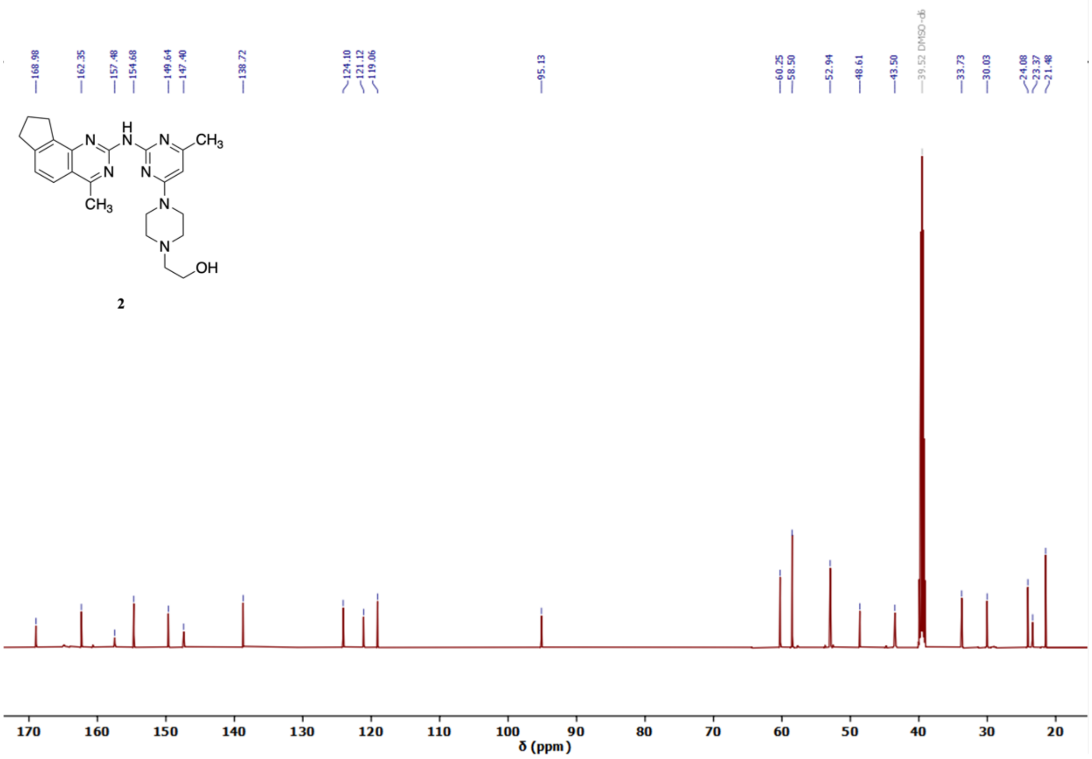

^{31}P NMR spectrum of **3** using H_3PO_4 as external standard at 162 MHz.

Compound 4

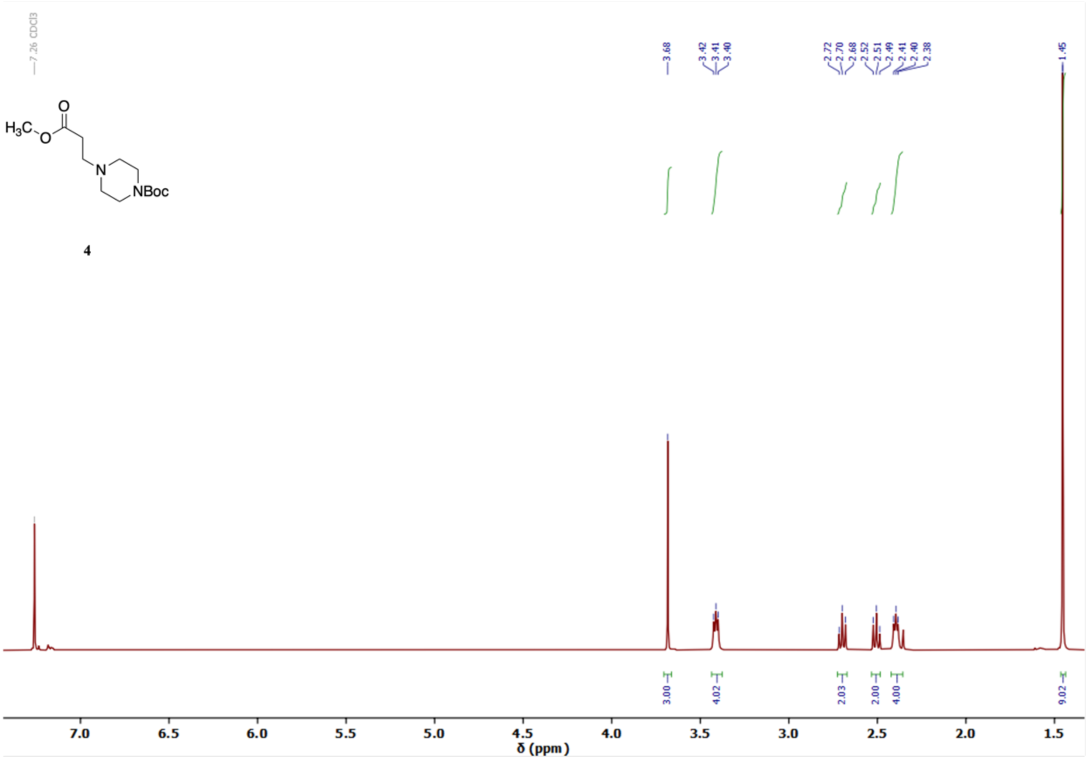

^{1}H NMR spectrum of **4** in chloroform-*d* measured at 400 MHz.

Compound 5

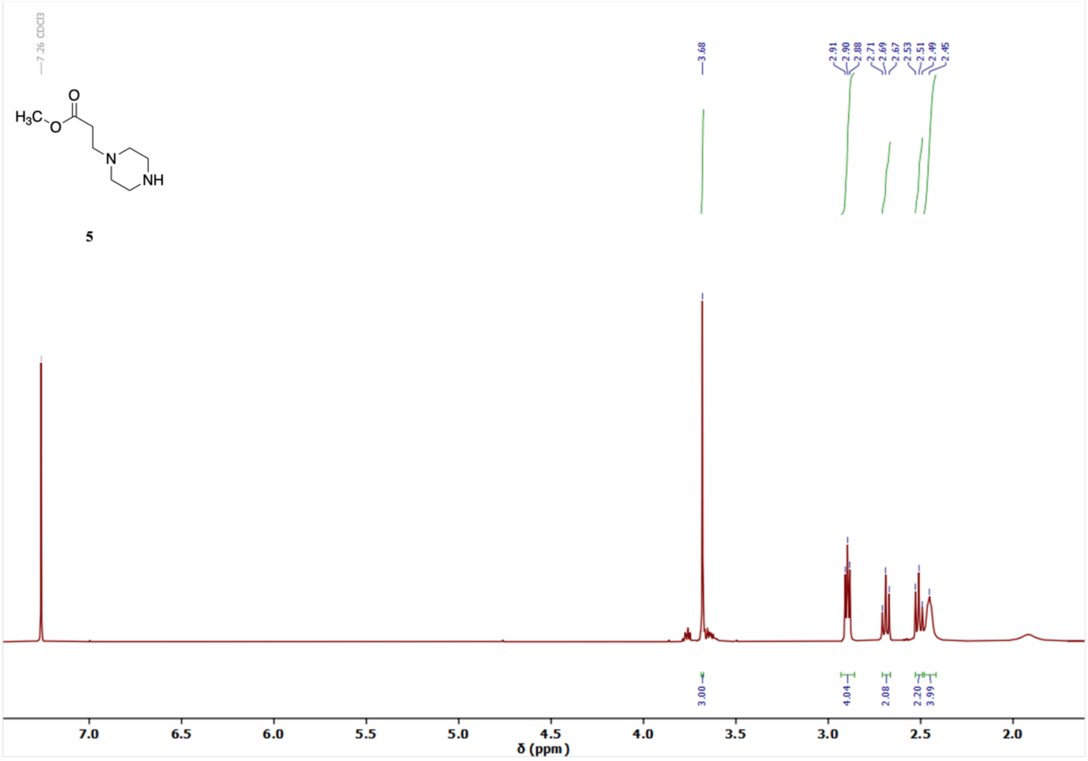

^{1}H NMR spectrum of **5** in chloroform-*d* measured at 400 MHz.

Compound 6

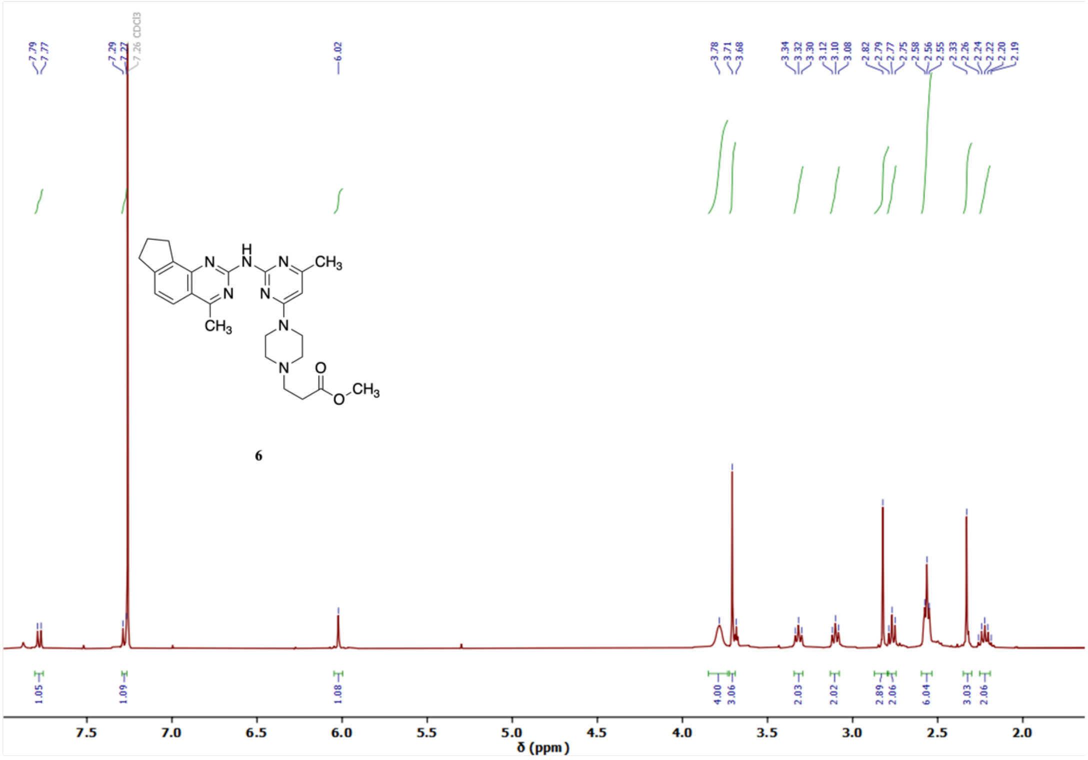

^{1}H NMR spectrum of **6** in chloroform-*d* measured at 400 MHz.

Compound 7

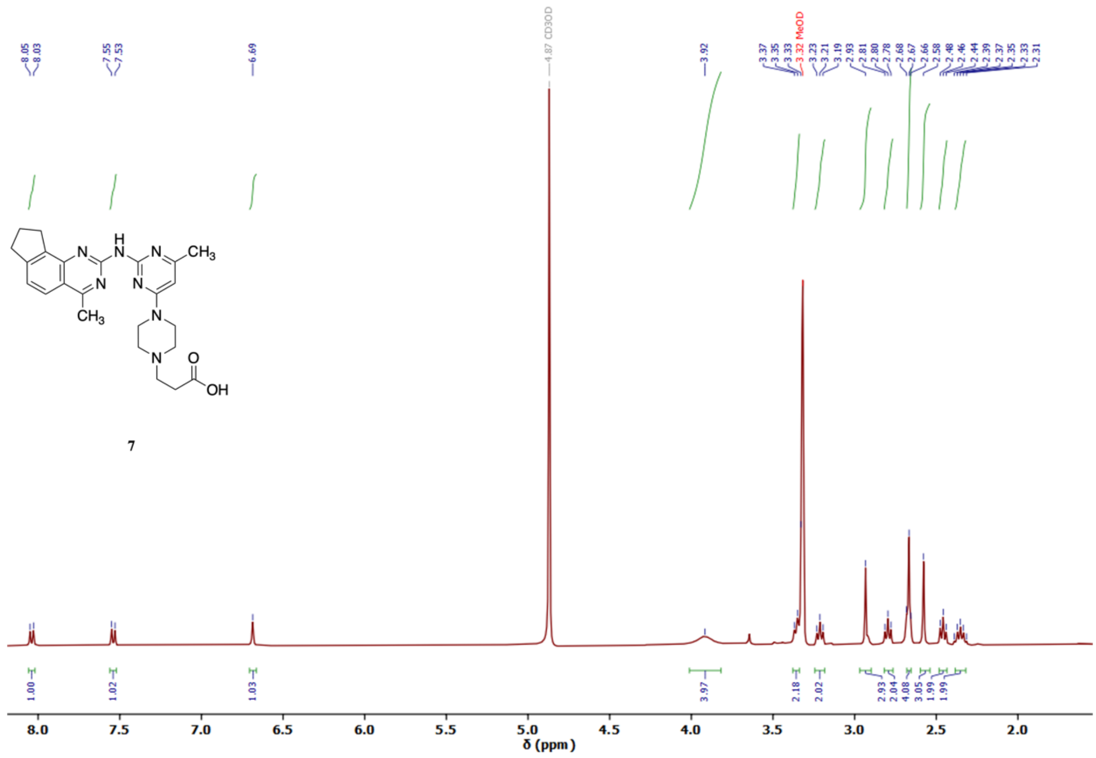

^{1}H NMR spectrum of **7** in methanol-d_4 measured at 400 MHz.

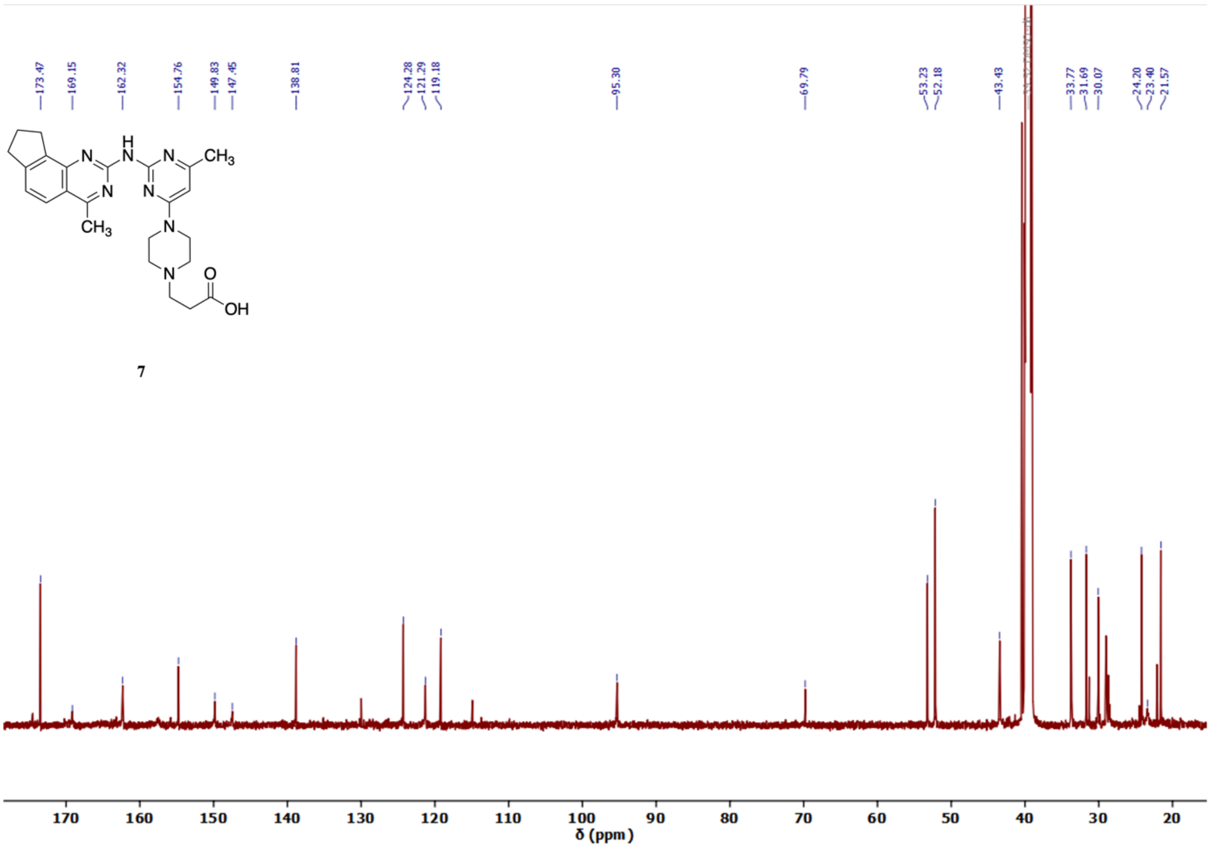

^{13}C NMR spectrum of **7** in DMSO-d_6 measured at 151 MHz.

Chromatograms of the GL-O conjugates

GL-O1

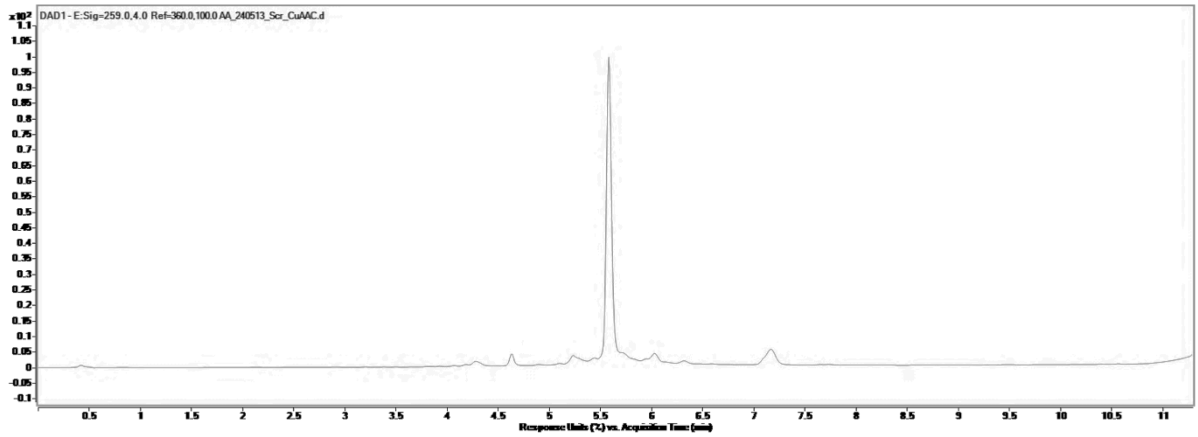

GL-O2

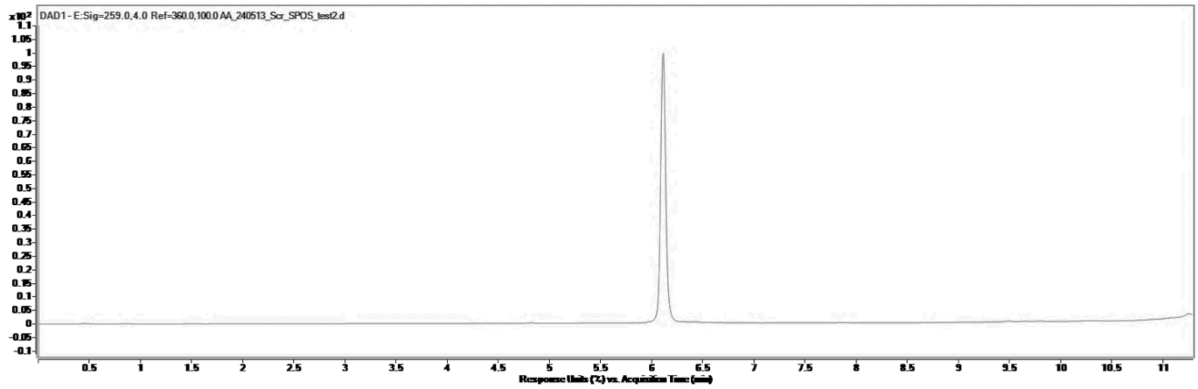

GL-O3

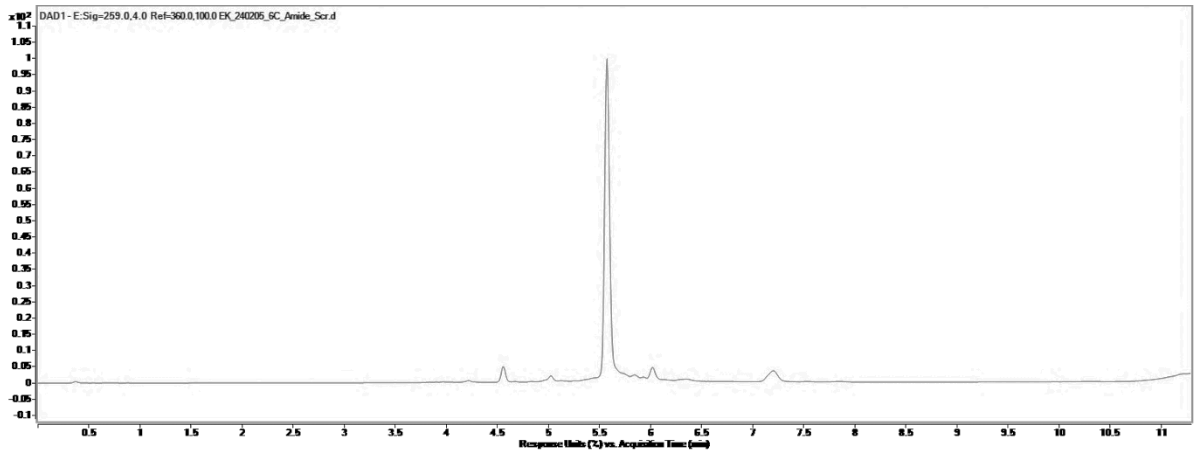

GL-O4

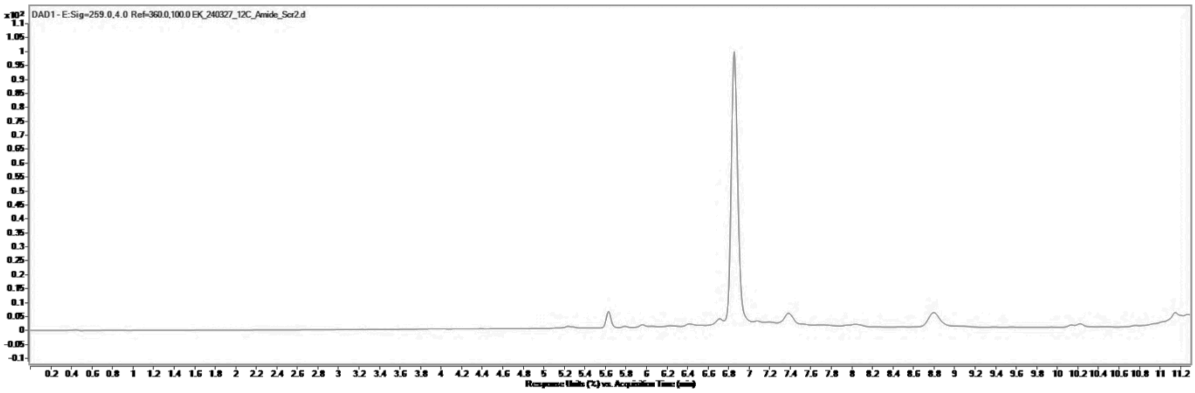

GL-O5

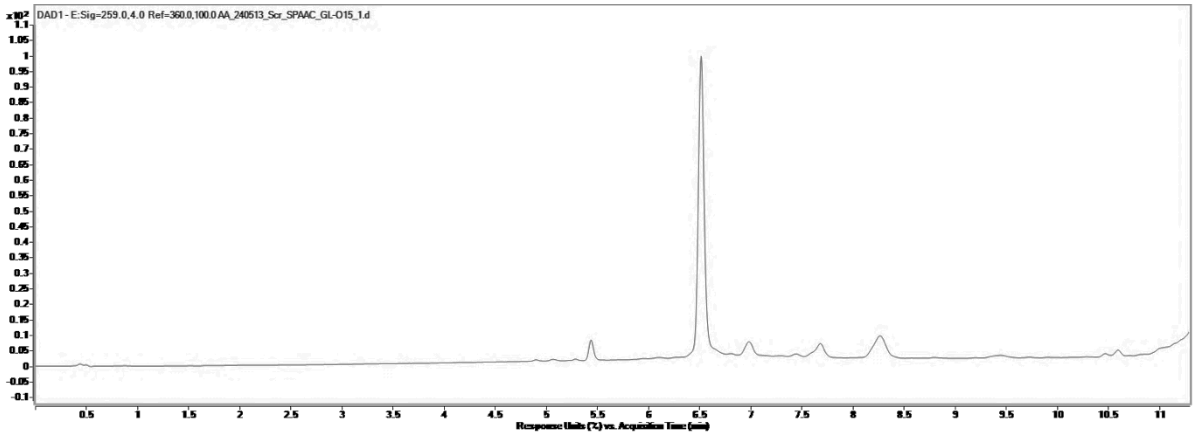

GL-O6

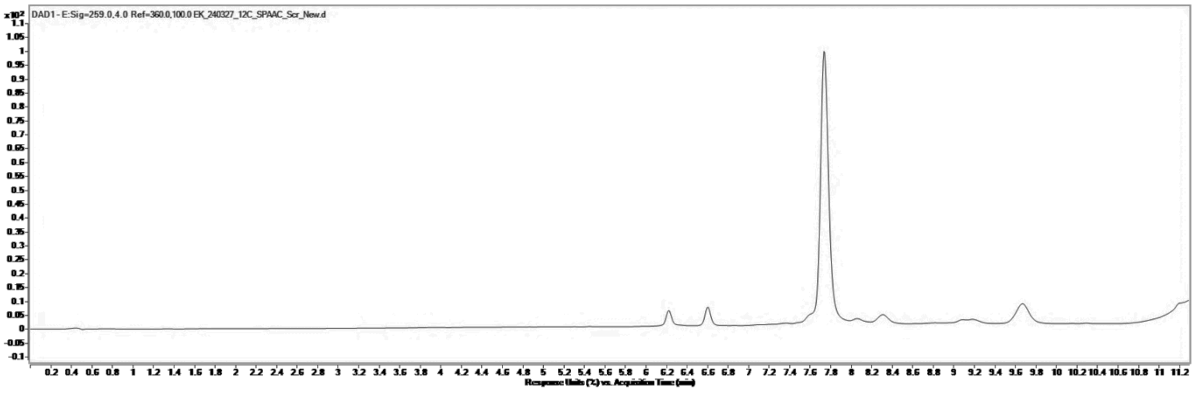

GL-O7

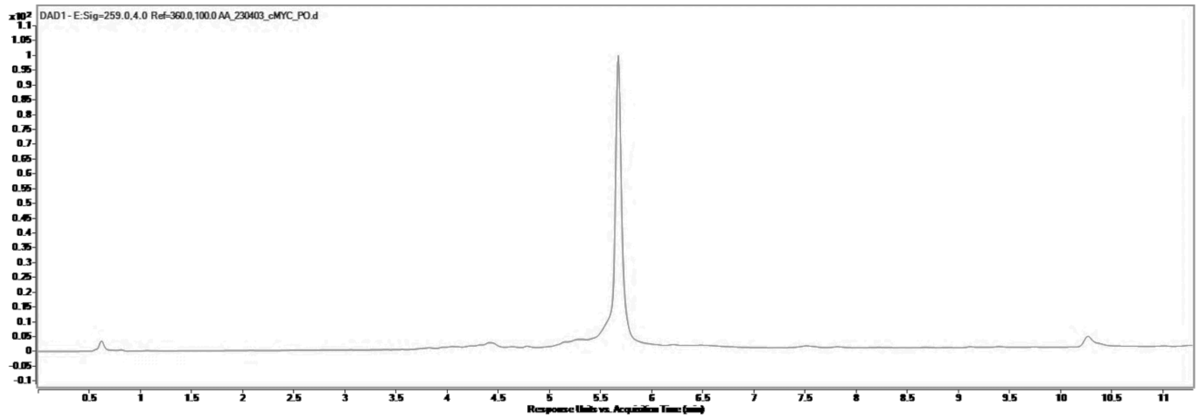

GL-O8

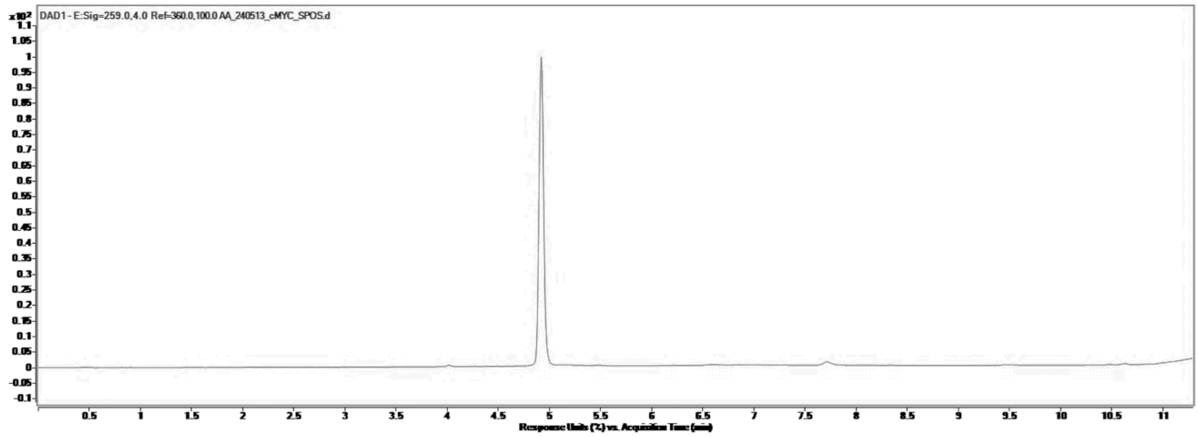

GL-O9

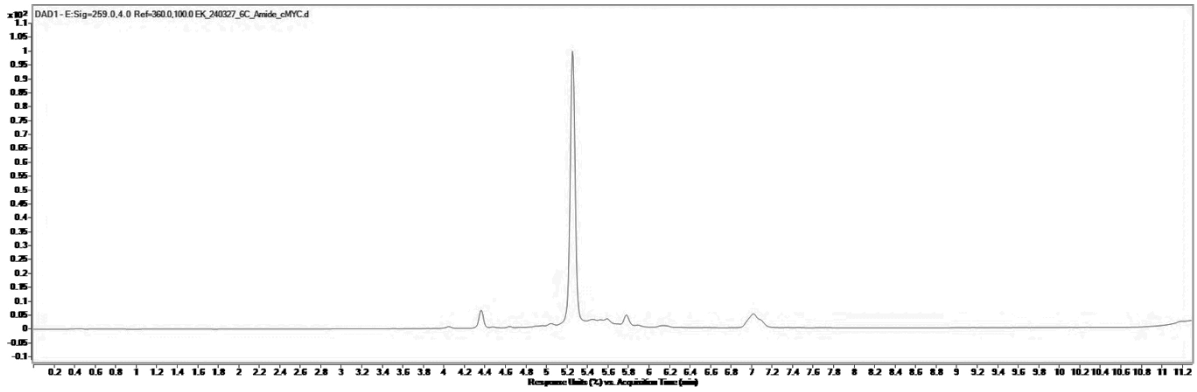

GL-O10

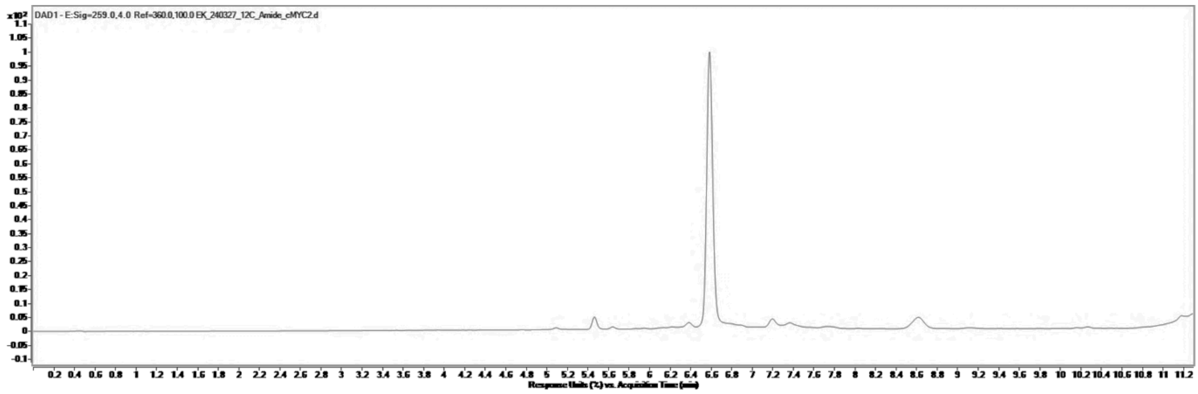

GL-O11

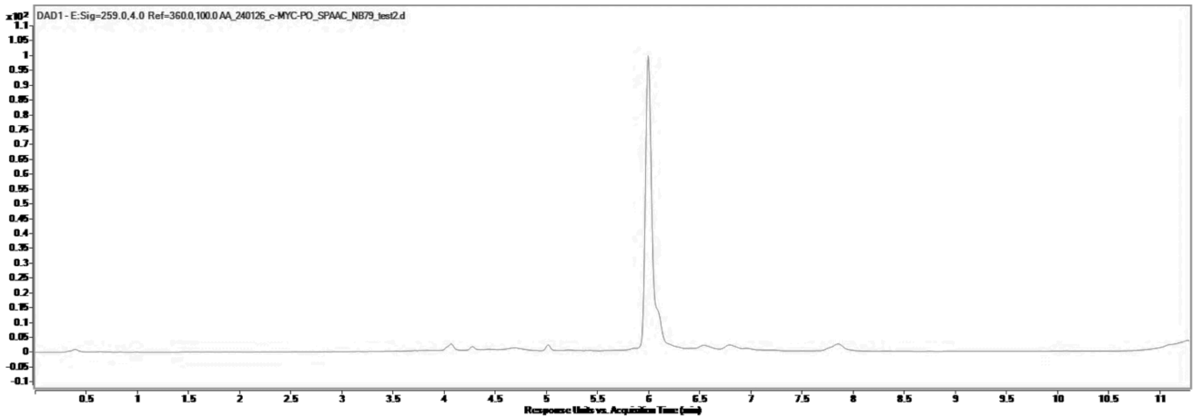

GL-O12

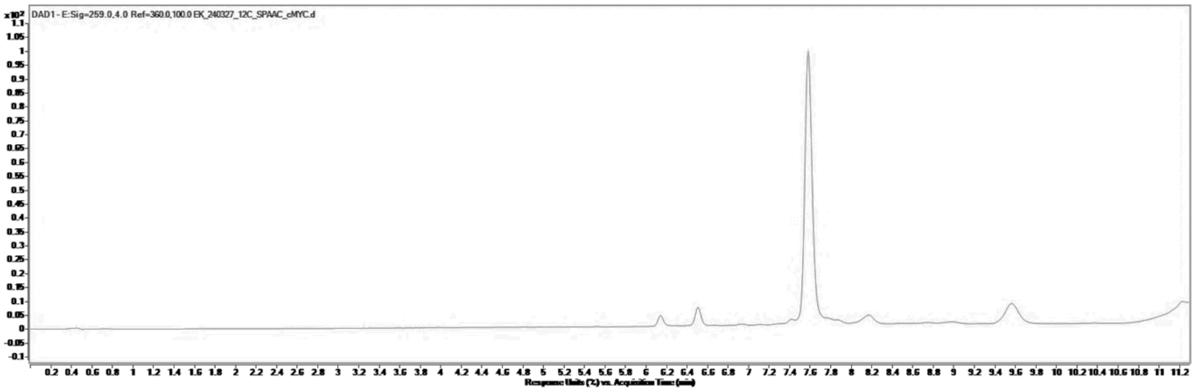

Table S3. Yields and mass of the GL-O conjugates.

Conjugate name	Yield	Calc. MS (g/mol)	Found MS (m/z)
**GL-O1**	76	5116.65	5116.06
**GL-O2**	23	4990.99	4990.99
**GL-O3**	38	5120.67	5120.09
**GL-O4**	58	5204.84	5204.14
**GL-O5**	70	5311.91	5311.11
**GL-O6**	76	5396.07	5395.20
**GL-O7**	15	5062.60	5062.00
**GL-O8**	11	4936.98	4936.98
**GL-O9**	24	5066.63	5065.97
**GL-O10**	49	5150.79	5150.13
**GL-O11**	50	5257.86	5257.11
**GL-O12**	23	5342.02	5341.20

In vitro assays

Microscale thermophoresis (MST). The G4 template consisting of the G4-forming sequence and the flanking sequence was purchased with a Cy5 5´-label. The G4 DNA was annealed in MST buffer (10 mM potassium phosphate, 100 mM KCl, 0.05% Tween 20, pH 7.4) by heating at 96 °C for 5 min followed by cooling down to room temperature before storing in the fridge overnight. All MST experiments were done on a Monolith NT.115 (Nanotemper, Germany) instrument and performed in MST buffer with standard Monolith capillaries. The G4 DNA concentration was kept constant at 10-20 nM and the GL-Os were serial diluted (1:1) with highest concentration of 5-20 µM. MST traces and binding affinity constants (K_D) were obtained using the Monolith analysis software and plotted and visualized in GraphPad Prism 10.

Taq polymerase STOP assay. Taq Polymerase stop assay was adapted from Jamroškovič et al.[1] DNA templates were annealed to fluorescent labelled primers in 100 mM KCl by heating to 95 °C for 5 minutes followed by slow cooling to room temperature. The indicated compound concentrations were added to 40 nM annealed template in 1x Taq Buffer (10 mM Tris-HCl pH 8.8, 50 mM KCl, Thermo Fisher Scientific), 1.5 mM $MgCl_2$, and 0.05 U/µL Taq Polymerase (Thermo Fisher Scientific). Samples were preincubated on ice (10 minutes) and reactions initiated with the addition of dNTPS (100 µM) and transferring the samples to 37 °C. After 15 min at 37 °C, reactions were stopped by addition of equal volume of 2x stop solution (0.5% SDS, 25 mM EDTA, XC-Dye in Formamide) and separated on a 12% polyacrylamide Tris-Borate-EDTA (TBE) gel containing 25% formamide and 8 M urea. Fluorescent signal was detected with a Typhoon Scanner (Amersham Biosciences). The intensity of the full-length band was quantified using Image Quant TL 10.2 software (GE Healthcare Life Sciences) and compared to sample without compound.

NMR and thermal stability assay. A G4 DNA solution was annealed in 10 mM potassium phosphate buffer (3 mM KCl, 110 µM G4 DNA, pH 7.4) by heating at 96 °C for 5 min followed by cooling down slowly to room temperature and stored in the fridge overnight. To the G4 DNA solution (180 µL), D_2O (20 µL) was added to yield a 100 µM G4 DNA solution which was transferred to a 3 mm NMR tube. GL-O, GL or oligo (1 mM in H_2O) was added (2 µL, 0.5 equiv.) and after 10 min, ^1H NMR spectrum was recorded. This was repeated one more time with an additional 0.5 equiv. GL-O to yield a 1:1 molar ratio with G4 DNA. A 850 MHz Avance III HD spectrometer equipped with a 5 mm TCI cryoprobe at 298 K was used for the experiments. Transmitter frequency offset (O1P) was set at 4.7 ppm and spectral width (SW) was fixed as 22 ppm. Excitation sculpting was used in the 1D ^1H experiments, and 512 scans were used to record the spectra. For the thermal stability assay, the samples were equilibrated at temperatures ranging from 313-338 K for 5 min followed by recording of the spectra every 2.5-10 K interval. All data were processed in MestreNova 10.0.2.

Computational modelling

Preparation of atom coordinates of G4 in complex with oligonucleotide. The models of G4s with varying gap lengths in complex with an oligonucleotide was based on an NMR structure of G4 (PDB:2MGN[3]). The structure was prepared using the protein preparation wizard[4, 5] implemented in the Schrödinger package. The co-crystallized ligand was removed, bond orders were assigned and hydrogens added at pH 7.0. Missing nucleotides were then manually added according to the sequence presented in Table S1. The resulting model was minimized using Macromodel within the Schrödinger package, with constraints on heavy atoms using a force constant of 100 kJ/mol/Å and the half-width of a flat-bottomed restraint set to 0.2 Å. The conformational space of the resulting models was thereafter investigated using molecular dynamics (MD) simulations.

Molecular Dynamics Simulations. The pdb2gmx within the GROMACS[6] package was used for generation of topology and coordinate files for the G4s in complex with an oligonucleotide. The Amber99 force field with PARMBSC1[7] was used. Solvation was done by addition of TIP3P water[8] molecules as a dodecahedron periodic box followed by neutralization by addition of counter ions with an excess of 0.1 M KCl. The GROMACS 2016 simulation package was used for running MD simulations. Energy minimization was performed using the steepest descent algorithm for removal of atomic clashes. Heating of the system from 0 to 300 K during 100 ps NVT simulations was performed, followed by equilibration of densities at 1 atm pressure during 500 ps NPT simulations. Heavy atoms were restrained at their starting positions using a force constant of 1000 kJ mol^{-1} nm^{-2}, and the restraints were linearly removed during a subsequent 1 ns NPT simulation. Temperature and pressure were regulated using the Berendsen algorithm.[9] 5×100 ns production simulations were performed using random initial velocities, with the temperature and pressure maintained at 300 K and 1 atm with 0.1 ps and 1 ps time constants using the v rescale temperature and the Parrinello-Rahman pressure coupling method,[10, 11] respectively. Short range non-bonded interactions were computed for atom pairs within a 14 Å distance. Long-range electrostatic interactions were calculated using the Particle-Mesh- Ewald summation method with fourth-order cubic interpolation with a 1.2 Å grid spacing.[12] Time steps during the simulations were 2 fs with bonds constrained using the parallel LINCS algorithm.[13, 14]

Trajectory Analysis. *RMSD*. Superpositioning of the backbone atoms of the structure towards the NPT simulated structure was performed followed by calculation of root mean-square deviation (RMSD) values using the gmx rms module in GROMACS. Based on the RMSD values (Figure S11b-d), the first 20 ns from each trajectory was discarded and remaining trajectories were concatenated and used for subsequent analyses.

PCA and clustering. The trajectory was superposed against the NPT simulated starting structure, using the backbone atoms of the G4 structure only (5'-3': GGG TGG TGA GGG TGG GGA AGG). The mass-weighted covariance matrix was calculated for the backbone atoms and diagonalized using gmx govar and the resulting eigenvectors were analyzed using gmx anaeig. The trajectory was projected onto the first three eigenvectors, (principal components (PCs)). Representative conformations of the PCA subspace were thereafter extracted through cluster analysis performed with gmx_clusterByFeatures[15] using PC1-3 as features (Figure S12-14). The K-means algorithm[16] was used and the number of clusters were determined using the Elbow method with a threshold of 2.5% applied on the Sum of Square Residual (SSR) to Sum of Square Total (SST) ratio. The centroid structure of each cluster was extracted for visualization, and the population of each cluster is presented in Table S4. Centroids of selected clusters were thereafter used for modelling of the GL-Os in complex with G4s.

Table S4. Population of the extracted clusters for the G4s in complex with an oligonucleotide.

Cluster	Population (%)		
	**1nt**	**3nt**	**9nt**
**C1**	24	20	23
**C2**	22	20	19
**C3**	22	20	19
**C4**	13	14	17
**C5**	12	13	11
**C6**	7	11	6
**C7**	-	2	5

Docking of ligands. The receptor grid generation tool implemented in the Schrödinger package was used to prepare a grid for docking. Only the G4 structure (5'-3': GGG TGG TGA GGG TGG GGA AGG) of selected structures from the MD trajectories was included. The center of the grid was based on the

position of nucleotides G4, G8, G13, G17 (numbering from PDB: 2MGN), with inner and outer box dimensions of 10×10×10 Å and 36×36×36 Å, respectively. Docking of ligands was performed with Glide[17-20] using standard precision (SP) mode, performing post docking minimization for 500 poses/ligand and thereafter collecting 50 poses/ligand. These resulting 50 poses were visualized, and one pose/ligand was selected based on their position in respect to the oligonucleotide it would be linked to.

Modelling of final G4s in complex with GL-Os. Selected linkers were manually added to the G4 oligonucleotide structure with docked ligands using the build tool in Maestro.[21] The resulting models were minimized using Macromodel[22-24] within the Schrödinger package in three steps using no solvent with constraints on heavy atoms, a force constant of 100 kJ/mol/Å, and the half-width of a flat-bottomed restraint set to 0.2 Å. During step 1, the linker and the nucleotides of the gap were allowed to move freely. During step 2, the linker, the nucleotides of the gap, and the ligand were allowed to move freely. During step 3 constraints were added to all heavy atoms. Thereafter, an energy minimization of the whole system was performed, without constraints, using water as solvent.

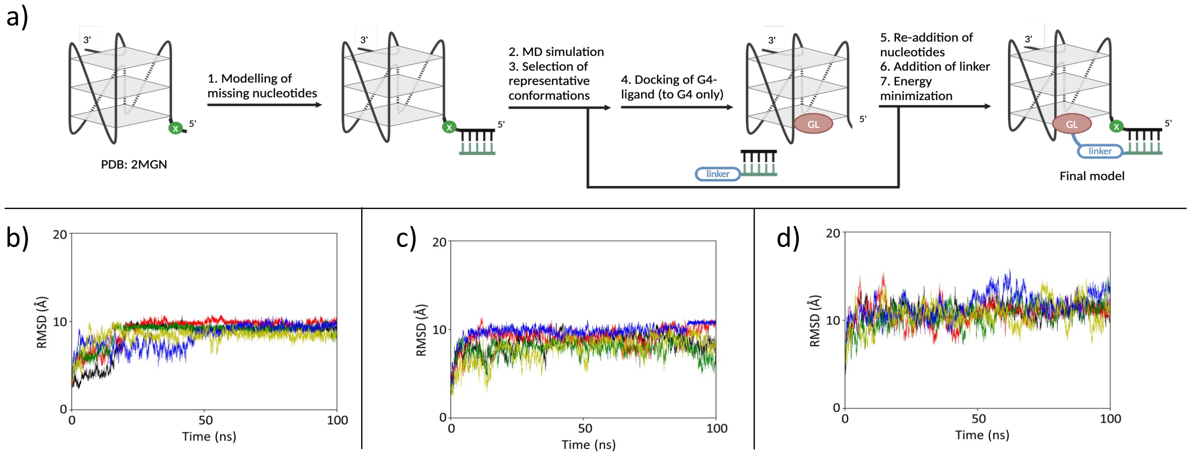

Figure S11. a) Overview of the modelling workflow of G4 DNA in complex with GL-Os. Root means square deviation (RMSD) values over the simulation time for the five MD simulations vs heavy atoms of G4s in complex with oligonucleotide. b) **1nt** c) **3nt** d) **9nt**.

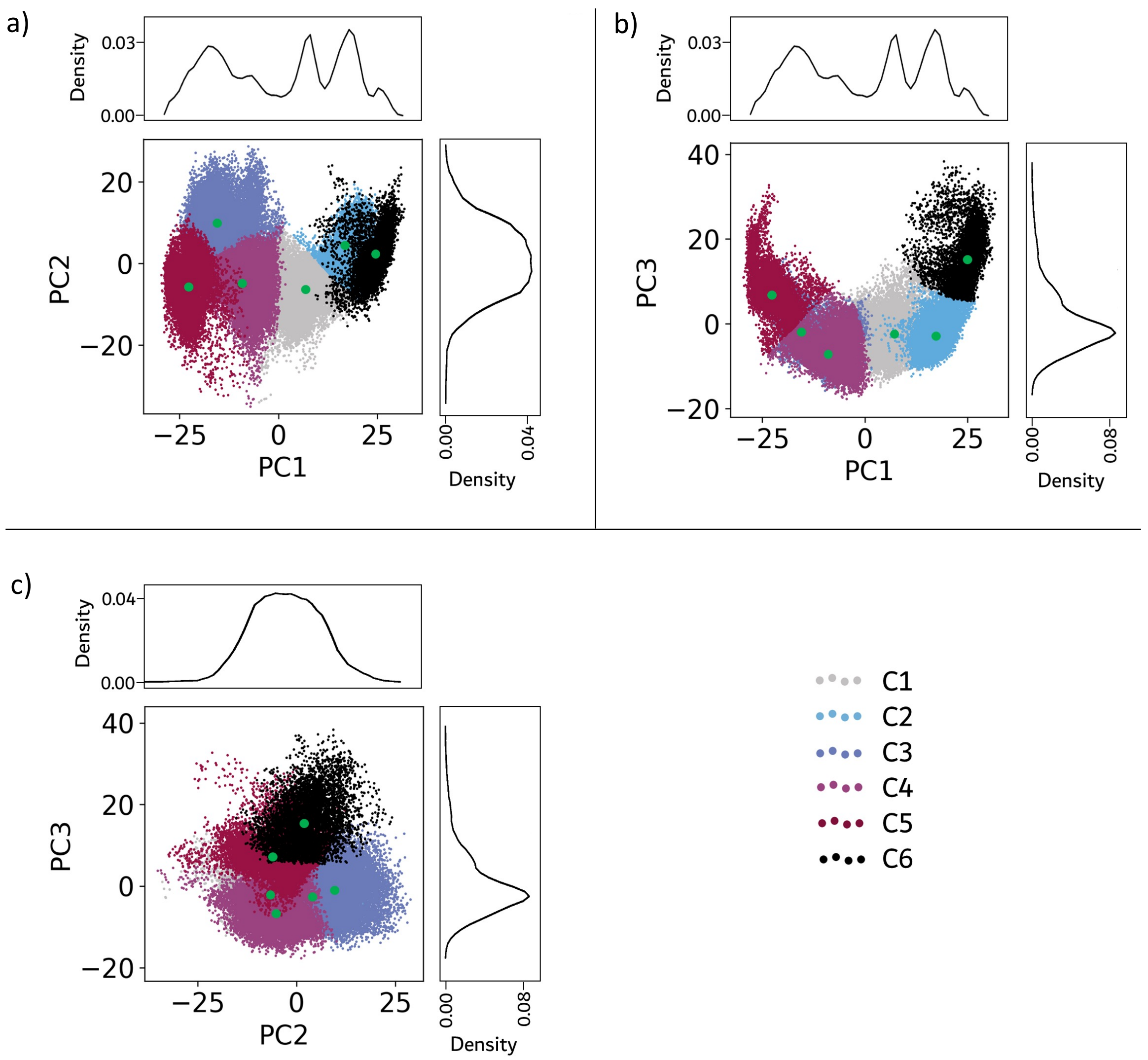

Figure S12. Projections of PC1-PC3 (A-C) for the combined trajectories of the G4 in complex with an oligonucleotide, G4 DNA template **1 nt**. The PCA was performed on the heavy atoms of the complete complex, after superposing against the heavy atoms of the G4 structure only (5'-3': GGG TGG TGA GGG TGGGGA AGG). The distribution of cluster 1-6 over the PCA subspace is represented by different colours with each dot representing one conformation. The green dots indicate the position of the central structure of each cluster. The histograms show the relative number of conformations in the PCA subspace. The largest, second largest, and third largest collective motion of the complex over the simulation time can be seen along PC1, PC2, and PC3, respectively.

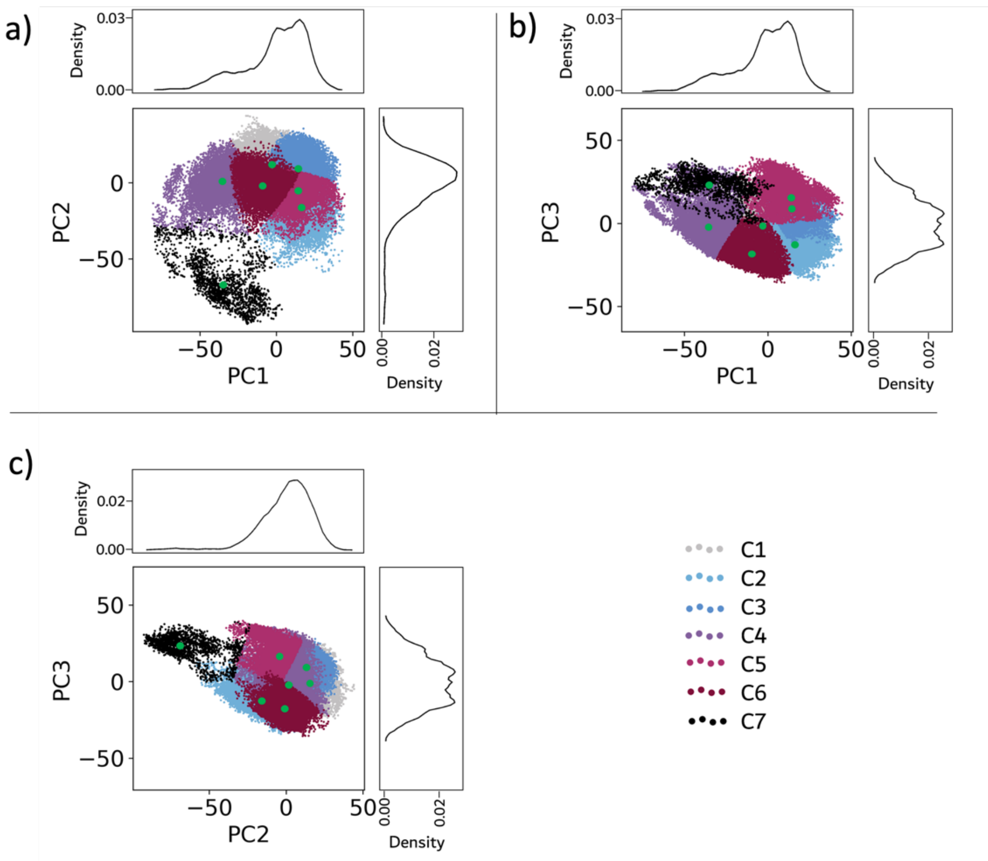

Figure S13. Projections of PC1-PC3 (A-C) for the combined trajectories of the G4 in complex with an oligonucleotide, G4 DNA template **3 nt**. The PCA was performed on the heavy atoms of the complete complex, after superposing against the heavy atoms of the G4 structure only (5'-3': GGG TGG TGA GGG TGG GGA AGG). The distribution of cluster 1-7 over the PCA subspace is represented by different colours with each dot representing one conformation. The green dots indicate the position of the central structure of each cluster. The histograms show the relative number of conformations in the PCA subspace. The largest, second largest, and third largest collective motion of the complex over the simulation time can be seen along PC1, PC2, and PC3, respectively.

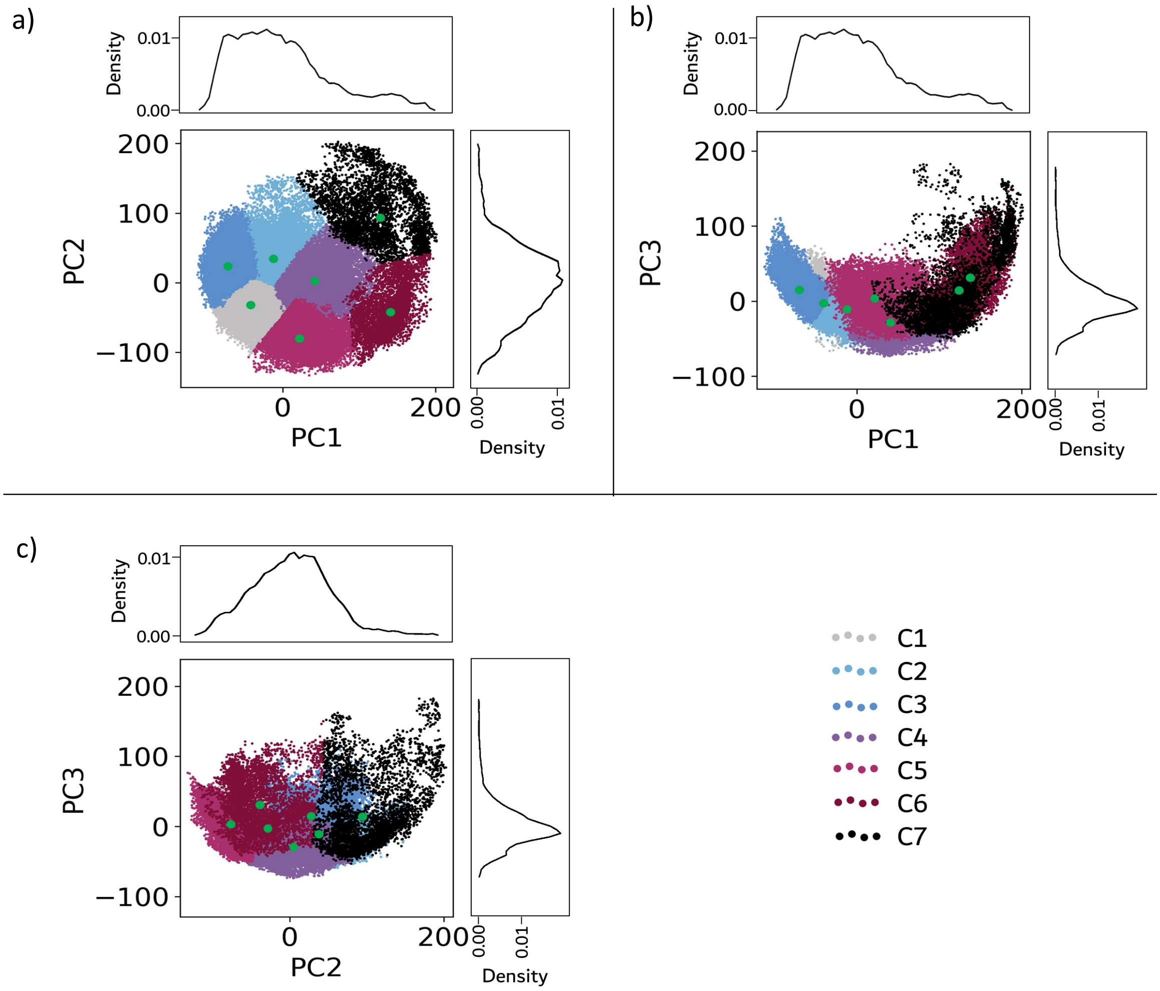

Figure S14. Projections of PC1-PC3 (A-C) for the combined trajectories of the G4 in complex with an oligonucleotide, G4 DNA template **9 nt**. The PCA was performed on the heavy atoms of the complete complex, after superposing against the heavy atoms of the G4 structure only (5'-3': GGG TGG TGA GGG TGG GGA AGG). The distribution of cluster 1-7 over the PCA subspace is represented by different colours with each dot representing one conformation. The green dots indicate the position of the central structure of each cluster. The histograms show the relative number of conformations in the PCA subspace. The largest, second largest, and third largest collective motion of the complex over the simulation time can be seen along PC1, PC2, and PC3, respectively.

References

(1) Berner, A.; Das, R. N.; Bhuma, N.; Golebiewska, J.; Abrahamsson, A.; Andréasson, M.; Chaudhari, N.; Doimo, M.; Bose, P. P.; Chand, K.; et al. G4-Ligand-Conjugated Oligonucleotides Mediate Selective Binding and Stabilization of Individual G4 DNA Structures. *J. Am. Chem. Soc.* **2024**, *146* (10), 6926-6935. DOI: 10.1021/jacs.3c14408.

(2) Bhuma, N.; Chand, K.; Andréasson, M.; Mason, J.; Das, R. N.; Patel, A. K.; Öhlund, D.; Chorell, E. The effect of side chain variations on quinazoline-pyrimidine G-quadruplex DNA ligands. *Eur. J. Med. Chem.* **2023**, *248*, 115103. DOI: 10.1016/j.ejmech.2023.115103.

(3) Chung, W. J.; Heddi, B.; Hamon, F.; Teulade-Fichou, M.-P.; Phan, A. T. Solution Structure of a G-quadruplex Bound to the Bisquinolinium Compound Phen-DC3. *Angew. Chem. Int.Ed.* **2014**, *53* (4), 999-1002. DOI: 10.1002/anie.201308063.

(4) Madhavi Sastry, G.; Adzhigirey, M.; Day, T.; Annabhimoju, R.; Sherman, W. Protein and ligand preparation: parameters, protocols, and influence on virtual screening enrichments. *J. Comput. Aided Mol. Des.* **2013**, *27* (3), 221-234. DOI: 10.1007/s10822-013-9644-8.

(5) Protein Preparation Wizard; Epik, Schrödinger; Schrödinger: New York, 2022.

(6) Pronk, S.; Páll, S.; Schulz, R.; Larsson, P.; Bjelkmar, P.; Apostolov, R.; Shirts, M. R.; Smith, J. C.; Kasson, P. M.; van der Spoel, D.; et al. GROMACS 4.5: a high-throughput and highly parallel open source molecular simulation toolkit. *Bioinform.* **2013**, *29* (7), 845-854. DOI: 10.1093/bioinformatics/btt055.

(7) Lindahl, V.; Villa, A.; Hess, B. Sequence dependency of canonical base pair opening in the DNA double helix. *PLOS Comput. Biol.* **2017**, *13* (4), e1005463. DOI: 10.1371/journal.pcbi.1005463.

(8) Jorgensen, W. L.; Chandrasekhar, J.; Madura, J. D.; Impey, R. W.; Klein, M. L. Comparison of simple potential functions for simulating liquid water. *J. Chem. Phys.* **1983**, *79* (2), 926-935. DOI: 10.1063/1.445869.

(9) Berendsen, H. J. C.; Postma, J. P. M.; van Gunsteren, W. F.; DiNola, A.; Haak, J. R. Molecular dynamics with coupling to an external bath. *J. Chem. Phys.* **1984**, *81* (8), 3684-3690. DOI: 10.1063/1.448118.

(10) Bussi, G.; Donadio, D.; Parrinello, M. Canonical sampling through velocity rescaling. *J. Chem. Phys.* **2007**, *126* (1). DOI: 10.1063/1.2408420.

(11) Nosé, S.; Klein, M. L. Constant pressure molecular dynamics for molecular systems. *Mol. Phys.* **1983**, *50* (5), 1055-1076. DOI: 10.1080/00268978300102851.

(12) Darden, T.; York, D.; Pedersen, L. Particle mesh Ewald: An N·log(N) method for Ewald sums in large systems. *J. Chem. Phys.* **1993**, *98* (12), 10089-10092. DOI: 10.1063/1.464397.

(13) Hess, B. P-LINCS: A Parallel Linear Constraint Solver for Molecular Simulation. *J. Chem. Theory Comput.* **2008**, *4* (1), 116-122. DOI: 10.1021/ct700200b.

(14) Hess, B.; Bekker, H.; Berendsen, H. J. C.; Fraaije, J. G. E. M. LINCS: A linear constraint solver for molecular simulations. *J. Comput. Chem.* **1997**, *18* (12), 1463-1472. DOI: 10.1002/(SICI)1096-987X(199709)18:12<1463::AID-JCC4>3.0.CO;2-H.

(15) https://github.com/rjdkmr/gmx *clusterByFeatures.* (accessed December 2024).

(16) Lloyd, S. Least squares quantization in PCM. *IEEE Trans. Inf. Theory* **1982**, *28* (2), 129-137. DOI: 10.1109/TIT.1982.1056489.

(17) Yang, Y.; Yao, K.; Repasky, M. P.; Leswing, K.; Abel, R.; Shoichet, B. K.; Jerome, S. V. Efficient Exploration of Chemical Space with Docking and Deep Learning. *J. Chem. Theory Comput.* **2021**, *17* (11), 7106-7119. DOI: 10.1021/acs.jctc.1c00810.

(18) Halgren, T. A.; Murphy, R. B.; Friesner, R. A.; Beard, H. S.; Frye, L. L.; Pollard, W. T.; Banks, J. L. Glide: A New Approach for Rapid, Accurate Docking and Scoring. 2. Enrichment Factors in Database Screening. *J. Med. Chem.* **2004**, *47* (7), 1750-1759. DOI: 10.1021/jm030644s.

(19) Friesner, R. A.; Banks, J. L.; Murphy, R. B.; Halgren, T. A.; Klicic, J. J.; Mainz, D. T.; Repasky, M. P.; Knoll, E. H.; Shelley, M.; Perry, J. K.; et al. Glide: A New Approach for Rapid, Accurate Docking and Scoring. 1. Method and Assessment of Docking Accuracy. *J. Med. Chem.* **2004**, *47* (7), 1739-1749. DOI: 10.1021/jm0306430.

(20) Schrödinger Release 2022-1: Glide, Schrödinger, LLC, New York, NY, 2022.
(21) Schrödinger Release 2022-1: Maestro, Schrödinger, LLC, New York, NY, 2022.
(22) Mohamadi, F.; Richards, N. G. J.; Guida, W. C.; Liskamp, R.; Lipton, M.; Caufield, C.; Chang, G.; Hendrickson, T.; Still, W. C. Macromodel—an integrated software system for modeling organic and bioorganic molecules using molecular mechanics. *J. Comput. Chem.* **1990**, *11* (4), 440-467. DOI: 10.1002/jcc.540110405.
(23) Watts, K. S.; Dalal, P.; Tebben, A. J.; Cheney, D. L.; Shelley, J. C. Macrocycle Conformational Sampling with MacroModel. *J. Chem. Inf. Model.* **2014**, *54* (10), 2680-2696. DOI: 10.1021/ci5001696.
(24) Schrödinger Release 2024-4: Glide, Schrödinger, LLC, New York, NY, 2024.
